# Genetic contribution to disease-course severity and progression in the SUPER-Finland study, a cohort of 10,403 individuals with psychotic disorders

**DOI:** 10.1038/s41380-024-02516-6

**Published:** 2024-04-01

**Authors:** Anders Kämpe, Jaana Suvisaari, Markku Lähteenvuo, Tarjinder Singh, Ari Ahola-Olli, Lea Urpa, Willehard Haaki, Jarmo Hietala, Erkki Isometsä, Tuomas Jukuri, Olli Kampman, Tuula Kieseppä, Kaisla Lahdensuo, Jouko Lönnqvist, Teemu Männynsalo, Tiina Paunio, Jussi Niemi-Pynttäri, Kimmo Suokas, Annamari Tuulio-Henriksson, Juha Veijola, Asko Wegelius, SUPERFinland-Researchers, Anders Kämpe, Jaana Suvisaari, Markku Lähteenvuo, Willehard Haaki, Erkki Isometsä, Tuomas Jukuri, Olli Kampman, Tuula Kieseppä, Teemu Männynsalo, Tiina Paunio, Jussi Niemi-Pynttäri, Kimmo Suokas, Annamari Tuulio-Henriksson, Asko Wegelius, Aija Kyttälä, Ari Ahola-Olli, Auli Toivola, Benjamin Neale, Huei-yi Shen, Imre Västrik, Jari Tiihonen, Jarmo Hietala, Jouko Lönnqvist, Juha Veijola, Kaisla Lahdensuo, Katja Häkkinen, Mark Daly, Minna Holm, Noora Ristiluoma, Risto Kajanne, Steven E. Hyman, Tarjinder Singh, Aarno Palotie, Olli Pietiläinen, Mark Daly, Jacob Taylor, Kenneth S. Kendler, Aarno Palotie, Olli Pietiläinen

**Affiliations:** 1 Institute for Molecular Medicine Finland (FIMM), University of Helsinki, Helsinki, Finland; 2 Department of Molecular Medicine and surgery (MMK), Karolinska Institutet, Stockholm, Sweden; 3 National Institute for Health and Welfare, Department of Mental Health and Substance Abuse Services, Helsinki, Finland; 4 Department of Forensic Psychiatry, University of Eastern Finland School of Medicine, Niuvanniemi hospital, Kuopio, Finland; 5 Broad Institute, Stanley Center for Psychiatric Research, Cambridge, MA USA; 6 Massachusetts General Hospital, Analytic and Translational Genetics Unit, Boston, MA USA; 7 Department of Psychiatry, University of Turku, Turku, Finland; 8 Department of Psychiatry, University of Helsinki and Helsinki University Hospital, Helsinki, Finland; 9 Department of Psychiatry, Oulu University Hospital, Oulu, Finland; 10 Faculty of Medicine and Health Technology, Tampere University, Tampere, Finland; 11 Department of Psychiatry, The Wellbeing Services County of Ostrobothnia, Ostrobothnia, Finland; 12 Department of Clinical Sciences, Psychiatry, Umeå University, Umeå, Sweden; 13 Department of Clinical Medicine (Psychiatry), Faculty of Medicine, University of Turku, Turku, Finland; 14 Hospital District of Helsinki and Uusimaa, Helsinki, Finland; 15 University of Helsinki, Helsinki, Finland; 16 National Institute for Health and Welfare, Helsinki, Finland; 17 SleepWell Research Program, Faculty of Medicine, University of Helsinki, Helsinki, Finland; 18 Tampere University, Tampere, Finland; 19 Department of Psychiatry, Tampere University Hospital, Tampere, Finland; 20 Department of Psychology and Logopedics, Faculty of Medicine, University of Helsinki, Helsinki, Finland; 21 Department of Psychiatry, Research Unit of Clinical Neuroscience, University of Oulu, Oulu, Finland; 22 Broad Institute Harvard, Program in Medical and Population Genetics, Cambridge, MA USA; 23 Harvard Medical School, Department of Medicine, Boston, USA; 24 Virginia Institute of Psychiatric and Behavioral Genetics, Richmond, VA USA; 25 Medical College of Virginia/Virginia Commonwealth University, Department of Psychiatry, Richmond, VA USA; 26 Neuroscience Center, Helsinki Institute of Life Science, University of Helsinki, Helsinki, Finland; 27 Department of Clinical Neuroscience, Karolinska Institutet, Stockholm, Sweden

**Keywords:** Schizophrenia, Genetics

## Abstract

Genetic factors contribute to the susceptibility of psychotic disorders, but less is known how they affect psychotic disease-course development. Utilizing polygenic scores (PGSs) in combination with longitudinal healthcare data with decades of follow-up we investigated the contributing genetics to psychotic disease-course severity and diagnostic shifts in the SUPER-Finland study, encompassing 10 403 genotyped individuals with a psychotic disorder. To longitudinally track the study participants’ past disease-course severity, we created a psychiatric hospitalization burden metric using the full-coverage and nation-wide Finnish in-hospital registry (data from 1969 and onwards). Using a hierarchical model, ranking the psychotic diagnoses according to clinical severity, we show that high schizophrenia PGS (SZ-PGS) was associated with progression from lower ranked psychotic disorders to schizophrenia (OR = 1.32 [1.23–1.43], *p* = 1.26e-12). This development manifested already at psychotic illness onset as a higher psychiatric hospitalization burden, the proxy for disease-course severity. In schizophrenia (*n* = 5 479), both a high SZ-PGS and a low educational attainment PGS (EA-PGS) were associated with increased psychiatric hospitalization burden (*p* = 1.00e-04 and *p* = 4.53e-10). The SZ-PGS and the EA-PGS associated with distinct patterns of hospital usage. In individuals with high SZ-PGS, the increased hospitalization burden was composed of longer individual hospital stays, while low EA-PGS associated with shorter but more frequent hospital visits. The negative effect of a low EA-PGS was found to be partly mediated via substance use disorder, a major risk factor for hospitalizations. In conclusion, we show that high SZ-PGS and low EA-PGS both impacted psychotic disease-course development negatively but resulted in different disease-course trajectories.

## Introduction

Psychotic disorders display considerable sharing of clinical symptoms and underlying genetic factors [1–3]. Consequently, the diagnostic separation is not always clear-cut, and it is common that an individual receives several diagnoses for different psychotic disorders over a lifetime. Such diagnostic shifts are often thought to reflect disease progression and changes in disease severity [4–7]. To account for these diagnostic shifts and to determine the main-lifetime diagnosis, hierarchical diagnostic models that rank the psychotic disorders are commonly used [8, 9]. Family-based genetic risk scores have recently been associated with specific psychiatric disease trajectories [10], but little is still known about how genetic factors contribute to disease-course development in psychotic disorders.

Here we studied the genetic contribution to disease-course development in 10,403 genotyped individuals diagnosed with a psychotic disorder (ICD10 equivalents: F20-29, F31, F32.3, F33.3) and up to 50 years of retrospective follow-up from high quality, full coverage, nation-wide Finnish healthcare registries [11]. Utilizing data from the Finnish Hospital Discharge Registry, which started in 1969, we calculated a psychiatric hospitalization burden metric to track disease severity throughout the individual disease-courses of the study participants’. We combined the disease-course severity metric with polygenic scores (PGSs) and leveraged the directionality of the hierarchical diagnostic model used in the SUPER-Finland protocol [12, 13] to investigate the genetic contribution to psychotic diagnosis progression and disease-course severity. This innovative way to utilize the longitudinal Finnish in-hospital register, with 50 years of follow-up, and in combination with genetic data provides a novel way for partitioning the genetic contribution for relevant risk factors and disease course outcome.

## Methods

### Study population

The SUPER-Finland study [13] is part of the Stanley Global Neuropsychiatric Genetics Initiative and includes 10 403 individuals, with active consent, and at least one episode of psychotic illness (ICD10 equivalents: F20-F29, F31, F32.3 and F33.3). The study recruitment (2016–2018) was nation-wide with the aim of collecting a representative sample of individuals with psychotic disorder in Finland. The recruitment and assessment took in the majority of cases place in the individuals’ treating unit (psychiatric hospitals, psychiatric out-patient clinics, and primary care units). Extensive questionnaire and interview data was collected at study inclusion, which was performed by psychiatrists and nurses specifically hired for the SUPER project. The questionnaire focused on self-reported well-being (present), quality of life, sleep, substance use, smoking and current self-reported diagnoses, while the interview focused on sociodemographic information and earlier stages in life (e.g school performance and disruptive behavior in youth). Also, supervised cognitive assessments was performed using the Cambridge Neuropsychological Test Automated Battery (CANTAB). All information from study inclusion was obtained by medical professionals but the protocol did not include a structured diagnostic interview due to time limitations. Full details of the study protocol have recently been published [13]. The study was approved by the Ethics Committee of the Hospital District of Helsinki and Uusimaa (Reference number 202/13/03/00/15). All participants were 18 years or older at inclusion and gave written informed consent. Details in Supplementary methods and https://thl.fi/en/web/thl-biobank/for-researchers/sample-collections/super-study.

### Health care registry information

Information on specific diagnoses (ICD codes) and their time points were retrieved from the nation-wide Finnish National Care Register for Health Care and Register of Primary Health Care Visits (*thl.fi/en/web/thlfi-en/statistics-and-data/data-and-services/register-descriptions*). The clinical registry data includes information from (1) the hospital discharge registry (1969-); (2) specialized out-patient clinics (1998-) and (3) primary health care units (2011-) [Supplementary methods]. Because of the high quality of the Finnish health care registries [11], we only considered diagnoses recorded in the registries, and not self-reported diagnoses obtained at study inclusion. A main-lifetime psychotic diagnosis was set using a hierarchal diagnostic model contained within the SUPER-Finland study protocol [12, 13]. The model ranks the four major psychotic diagnosis based on clinical perception of severity as: (1) schizophrenia (SZ), *n* = 5 479; (2) schizoaffective disorder (SAD), *n* = 2124; (3) bipolar disorder (BD), *n* = 2 461; (4) major depressive disorder with psychotic features (psychotic MDD), *n* = 1542; (n refers to the number of individuals that ever received the diagnosis). In total, 8354 had a registry recorded diagnosis of at least one of these four major psychotic disorders, 1067 had unranked psychotic diagnoses and 405 had no registry-recorded psychotic diagnosis. These 405 individuals were treated for other psychiatric conditions and were included due to self-reported psychotic episodes, and/or due to misclassification at study inclusion [detailed in Supplementary methods, Table SM1 and Fig SM4]. Although the exact biological and clinical relationships between different psychotic disorders remain to be settled, similar hierarchical models have regularly been used, and on a group level SZ has been considered to be the top-ranking psychotic diagnosis [8, 9, 14]. SAD, although still a debated diagnosis, has often been viewed as an intermediate between BD and SZ [15–17], while psychotic MDD have been shown to be closest to BD [14].

### Endpoints and definitions

Given that the presence of a psychotic illness was the main criterion for inclusion in the SUPER-Finland study, the age of psychotic illness onset was defined as the first registry recorded diagnosis indicating a psychotic illness [ICD code definitions in Supplementary methods]. Similarly, BD was defined to include all ICD codes for BD without specifying current psychotic symptoms (ICD10 equivalents: F30.2|F31.X). Substance use disorder (SUD, *n* = 1 763) was defined as having had any recorded diagnosis indicating a substance abuse or substance misuse apart from nicotine dependence (ICD10 equivalents: F10-F16 and F18-F19). Self-reported cannabis usage was cross-sectionally recorded at study inclusion but was not used for defining a SUD-endpoint due to absence of longitudinal information.

### Psychiatric hospitalization burden

To track the study participants past disease-course severity a longitudinal metric was constructed based on the need for psychiatric hospital care using the Hospital Discharge Registry for which we had the longest follow-up (1969-01-01 to 2018-12-31). To account for changes in clinical practice over time and the steep decline in the number of psychiatric hospital beds during the last decades [18], the qualitative need for psychiatric in-patient care (hospital admissions of any length) for each individual and each year was recorded. The hospitalization data was then aligned for the first diagnosis of any psychotic disorder (set as the zero time point) to make the data comparable across individuals and take duration of illness into account. The hospitalization burden was calculated for each study participant as the fraction of years with at least one hospitalization primarily due to a psychiatric diagnosis for the first 15 years psychotic illness [detailed motivation for the construction of the metric in the Supplementary appendix]. To assess diagnostic progression, the hospitalization burden metric was used to assess the study participants’ disease severity for different timepoints during their disease-courses’ in relationship to their, at the time, current diagnoses.

### Genotyping and imputation

The study participants were all genotyped using the Illumina Global Screening Array. Samples with poor genotype quality, ancestral outliers and samples that mismatched with the recorded sex were excluded [Supplementary methods]. Imputation was performed using a Finnish specific reference panel SISu version 3 (*thl.fi/documents/3287543/3344176/THL+Biobank+Imputation+Panel.pdf*) and only reliably imputed variants (INFO score >0.8) were included. After quality control the study included 9 826 individuals.

### Construction of polygenic scores

Polygenic scores (PGSs), were constructed from the largest publicly available summary statistics at the time of publication for each trait of interest using MegaPRS [19] [Supplementary methods], which have shown good performance for psychiatric traits compared to other advanced PGS methods [20]. PGSs were constructed for seven psychiatric relevant traits: SZ, BD, major depressive disorder (MDD), intelligence, educational attainment (EA), CUD and alcohol dependence [2, 21–26].

### Statistical models

Cox regression and linear/logistic regression were used in the analyses. In the genetic association models, sex, year of birth and the 10 first principal components were used as covariates. Transformations and analysis specific covariates were used when appropriate [Supplementary methods]. The significance level was adjusted for multiple testing and indicated in all analyses. All statistical analyses and figures were generated using R 4.1.2. A graphical overview of the study’s analysis approach can be found in Fig S1.

## Results

### Genetic contribution to psychotic disorder progression

**Fig. 1:**
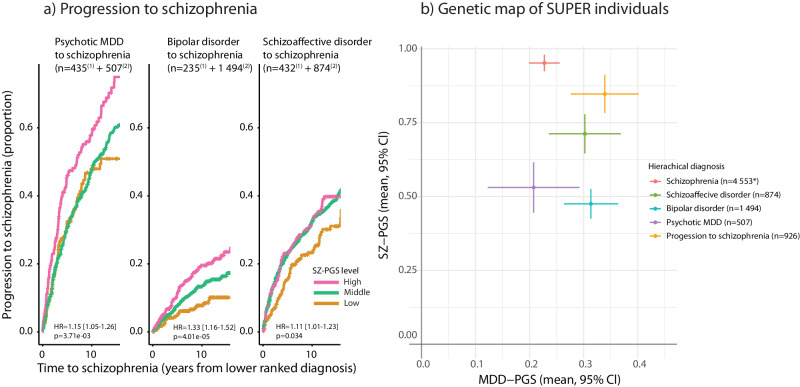
Progression to schizophrenia. **a** Progression from a lower ranked psychotic diagnosis to schizophrenia. The three panels show individuals who at some point in time had psychotic MDD, BD or SAD as their most severely ranked psychotic diagnosis but later progressed to schizophrenia compared to the individuals who remained at the corresponding lower ranked diagnoses. The results show that a larger proportion of individuals with a high SZ-PGS progressed to schizophrenia (combined HR = 1.23 [1.15-1.31], *p* = 6.42e-10). [SZ-PGS levels: Low = bottom <20%; Middle=20-60%; High = >80%; ^(1)^ Number of individuals who progressed to schizophrenia; ^(2)^ Number of individuals who remained at the specific lower ranked diagnosis.]. **b** Genetic map of SUPER participants. Polygenic composition of individuals with the four major psychotic diagnoses according to their hierarchical ranking, including the group that progressed to schizophrenia from an initial lower ranked diagnosis (orange). The psychotic and affective dimensions are proxied by the SZ-PGS and MDD-PGS respectively (mean PGS with error bars showing 95% Cl). The progression group displayed a higher SZ-PGS than any of the lower ranked diagnostic groups (*p* = 0.0178 (SAD), *p* = 3.06e-16 (BD) and *p* = 1.95e−07 (psychotic MDD)). [y-axis: SZ-PGS; x-axis: MDD-PGS; *=only individuals who had schizophrenia as their first major psychotic disorder].

To investigate the genetic contribution to the progression of psychotic disorders we leveraged a hierarchical model that ranked the four major psychotic disorders as: (1) SZ; (2) SAD; (3) BD and (4) psychotic MDD [“Methods”]. Of the study participants whose genotype data passed quality control, 8354 individuals had at least one of the four ranked major psychotic diagnoses recorded in the registries [“Methods”]. We defined diagnostic progression as a diagnostic shift from a lower ranked psychotic diagnosis to a higher ranked psychotic diagnosis. In total, 2605 individuals (31%) had received at least two of the four major psychotic diagnoses during their disease-course. The majority of these (*n* = 1561) had received the lowest ranked psychotic diagnosis first and most frequently had schizophrenia as their end-diagnosis (*n* = 926). To study diagnostic progression, we focused on these 926 individuals because they constituted the largest patient group with a uniform clinical endpoint (i.e progression from a lower ranked psychotic diagnosis to schizophrenia). We found that high SZ-PGS was associated with progression to schizophrenia from an initial lower ranked psychotic diagnosis (overall HR = 1.23 [1.15–1.31], *p* = 6.42e-10) [Fig. 1a]. The group that progressed to schizophrenia had on average a higher SZ-PGS than the lower ranked diagnostic groups (p = 0.0178 (SAD), *p* = 3.06e-16 (BD) and *p* = 1.95e-07 (psychotic MDD)) [Fig. 1b]. However, compared to the individuals who received schizophrenia as their first major psychotic diagnosis, the group that progressed to schizophrenia from a lower ranked diagnosis had a slightly lower SZ-PGS (mean difference: −0.10 SD, *p* = 0.0044). The lower ranked diagnostic groups (SAD, BD and psychotic MDD) characteristically present with more affective symptoms than in schizophrenia [27, 28], and the progression group did display a slightly higher MDD-PGS than other individuals with schizophrenia (mean difference: 0.11 SD, *p* = 0.0014).

### Psychiatric hospitalization burden captures disease severity and diagnostic progression within psychotic disorders

**Fig. 2:**
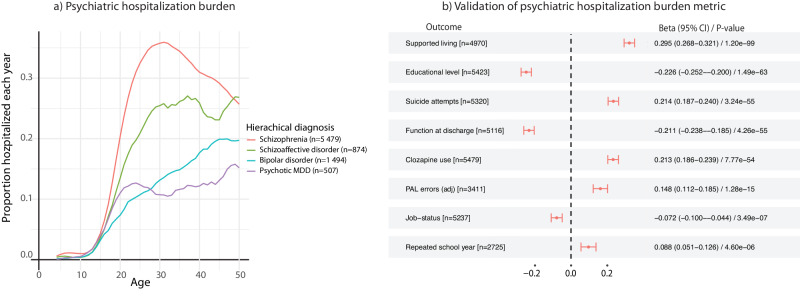
Psychiatric hospitalization burden. **a** The psychiatric hospitalization burden for the four major psychotic disorders according to the diagnostic hierarchical model, where the highest ranked psychotic disorder was considered the current lifetime diagnosis. The y-axis shows the proportion hospitalized due to a psychiatric diagnosis in each group and the x-axis shows the age at event. Individuals with schizophrenia had the highest overall burden compared to the lower ranking diagnoses (*β* = 0.65, *p* = 3.26e-186). **b** Associations between endpoints often thought to reflect general functioning and their association to the average yearly psychiatric hospitalization burden. The results show that an increased hospitalization burden was associated with a poor outcome for all endpoints, suggesting that the measurement can serve as a proxy to track disease-severity. Note, the analysis was constrained to individuals with schizophrenia (*n* = 5479) to not be influence by the cohort’s diagnostic composition. (Parameters have been named to make the direction of effect intuitive; n denotes the number of individuals with non-missing data for the specific endpoint; *Adjusted Paired Associates Learning (PAL) errors, a part of the CANTAB suit to assess cognitive function.).

Next, we investigated whether the disease-course severity of patients who progressed from a lower ranking psychotic disorder to schizophrenia differed from those who remained in their initial diagnostic group. For this purpose, we constructed a psychiatric hospitalization burden metric based on the yearly need for psychiatric hospital care as a method to track the participants past disease-course severity [“Methods”]. We observed that this metric conformed well with the hierarchical diagnostic severity ranking, where individuals with schizophrenia had the highest overall burden compared to the lower diagnostic groups [Fig. 2a]. Reassuringly, a high hospitalization burden showed consistently strong associations with outcomes often though to reflect general functioning [Fig. 2b], such as the need for supported housing and clozapine use, implying that the metric could serve as a proxy for disease-course severity.

We then compared the psychiatric hospitalization burden of the individuals that progressed to schizophrenia (*n* = 926) with those who remained at the lower ranked diagnoses (psychotic MDD, *n* = 507; BD, *n* = 1 494; SAD, *n* = 874). To understand how the hospitalization burden evolved from the start of the disease-course, we aligned the hospitalization data according to the age at onset of any psychotic disorder [“Methods”]. The comparison revealed that the individuals who later progressed to schizophrenia had a substantially higher hospitalization burden than individuals with lower ranked diagnoses. Moreover, already from the first recorded sign of psychosis, their hospitalization burden was as high as individuals who had schizophrenia as their first major psychotic diagnosis [Fig S2], despite having a delayed schizophrenia diagnosis (median delay = 5.6 years later, *p* = 1.02e-79) [Table 1]. The age at illness onset for the individuals that progressed to schizophrenia did not differ from the individuals who directly received a schizophrenia diagnosis (median difference: 0.1 years) but were significantly lower compared to individuals with any of the other three major psychotic diagnoses [Table 1]. This suggested that the overall severity of illness in the group that progressed to schizophrenia was similar to other individuals with schizophrenia already from disease-course start. The delay in schizophrenia diagnosis for the progression group was seen across decades, supporting the analysis strategy, and suggesting that the results are not a reflection of changing clinical diagnostic practices over time [Fig SA7, supplemental appendix]. Finally, we observed that compared to males, females were more likely to progress from a lower ranked diagnosis to schizophrenia than to receive schizophrenia as their first major psychotic disorder (OR = 1.72 [1.49–1.99], *p* = 1.08e-13) [Table 1], suggesting a potential diagnostic sex bias.

**Table 1:** Characteristics of the four major diagnostic groups compared to the group that progressed from lower ranked psychotic diagnoses to schizophrenia.

Diagnosis group	Age at psychotic onset (median/IQR)	Age at schizophrenia (median/IQR)	Delayed schizophrenia diagnosis (β/*p*-value)	Gender balance (Male/Female)
Schizophrenia (*n* = 4 553^a^)	24.4/20.4–30.0	26.4/21.7–33.3	–	59.2%/40.8%
Progression to schizophrenia (combined *n* = 926)	24.3/19.9–31.3	31.9/24.9–41.7	0.62/1.02e-79	46.9%/53.1%
Schizoaffective disorder (*n* = 874)	26.9/21.8–34.4	–	–	36.8%/63.2%
Schizoaffective disorder preceding schizophrenia (*n* = 432^b^)	24.3/20.1–31.5	35.3/26.4 - 45.3	0.73/2.23e-56	47.0%/53.0%
Bipolar disorder (*n* = 1 494)	34.0/25.0–43.7	–	–	37.3%/62.7%
Bipolar disorder preceding schizophrenia (*n* = 235^b^)	24.3/20.6–30.8	33.3/26.1–43.2	0.74/2.61e-33	46.8%/53.2%
Psychotic MDD (*n* = 507)	35.9/24.1–49.4	–	–	37.5%/62.5%
Psychotic MDD preceding schizophrenia (*n* = 435^b^)	24.5/19.3–32.3	30.3 / 23.4–39.8	0.58/5.87e-36	43.0%/57.0%

^a^Includes only individuals who had schizophrenia as their first major psychotic disorder.

^b^176 individuals were included in more than one of the above progression groups because they had received at least three of the major psychotic diagnoses at some point in time. However, the diagnostic hierarchy is unidirectional, meaning that a lower ranked diagnosis had to precede the more severely ranked diagnosis. In total 926 individuals progressed to schizophrenia form a lower ranked diagnosis.

### Genetic contribution to psychiatric hospitalization burden in schizophrenia

**Fig. 3:**
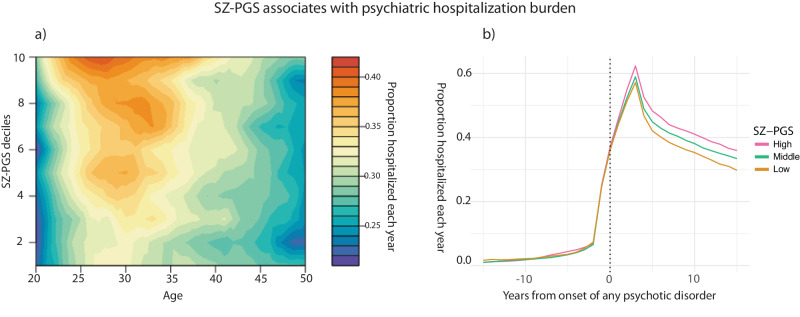
Hospital burden for individuals with schizophrenia. **a** Heatmap displaying the proportion hospitalized due to a psychiatric diagnosis each year (colored) in relationship to SZ-PGS deciles (y-axis) and age (x-axis). In individuals with schizophrenia a high SZ-PGS was associated with an increased need of psychiatric hospital care (*β* = 0.067, *p* = 1.58e-06). **b** The proportion hospitalized for a primary psychiatric diagnosis in relation to SZ-PGS. The hospitalization data was aligned for the time-point of first recorded psychotic illness. A high SZ-PGS was associated with an increased need of hospital care post psychotic illness onset (*β* = 0.054, *p* = 1.00e-04). [SZ-PGS strata: Low = <20% (*n* = 1 096); Middle = 20–60% (*n* = 3 287); High = >80% (*n* = 1 096); total *n* = 5 479].

We next investigated the genetic contribution to psychiatric hospitalization burden. Since the need for psychiatric hospital care were significantly different between the four specific psychotic diagnoses, we confined the analyses to individuals with schizophrenia (*n* = 5479) to avoid biases due to the study’s diagnostic composition. We evaluated the relationship between the yearly need of psychiatric hospitalization and PGSs for seven traits deemed relevant [“Methods”]. Among individuals with schizophrenia, the SZ-PGS was significantly associated with hospitalization burden over an individual’s adult life-course (*β* = 0.067, *p* = 1.58e-06). This was true also after aligning the data for the time-point of psychotic illness onset (*β* = 0.054, *p* = 1.00e-04) [Fig. 3a, b]. Because the underlying GWAS used to construct the SZ-PGS likely is enriched for treatment resistant cases, we also performed the analysis in non-clozapine users (*n* = 3377, *β* = 0.063, *p* = 4.81e-04) and observed that the association between the SZ-PGS and hospitalization burden remained virtually unchanged [2]. In addition to the SZ-PGS, the MDD-PGS also showed a strong influence on psychiatric hospitalization burden within schizophrenia individuals [Fig S3]. However, in contrast to high SZ-PGS, high MDD-PGS was associated with having been hospitalized for a non-psychotic depression and/or anxiety diagnoses (OR = 1.17 [1.11–1.24], *p* = 1.24e-07), which were common primary reasons for hospital admissions, both before and after the psychotic illness onset.

Intriguingly, the EA-PGS had the strongest association to psychiatric hospitalization burden following the onset of any psychotic disorder (*β* = −0.084, *p* = 4.53e-10) [Fig S3]. We took special interest in the EA-PGS because it was independent from the SZ-PGS (*r* = −0.017) [Fig S4], which suggested that the EA-PGS reflected a different mechanism of action that contributed to the need of psychiatric hospital care.

### The EA-PGS and the SZ-PGS displayed different hospital usage profiles

**Fig. 4:**
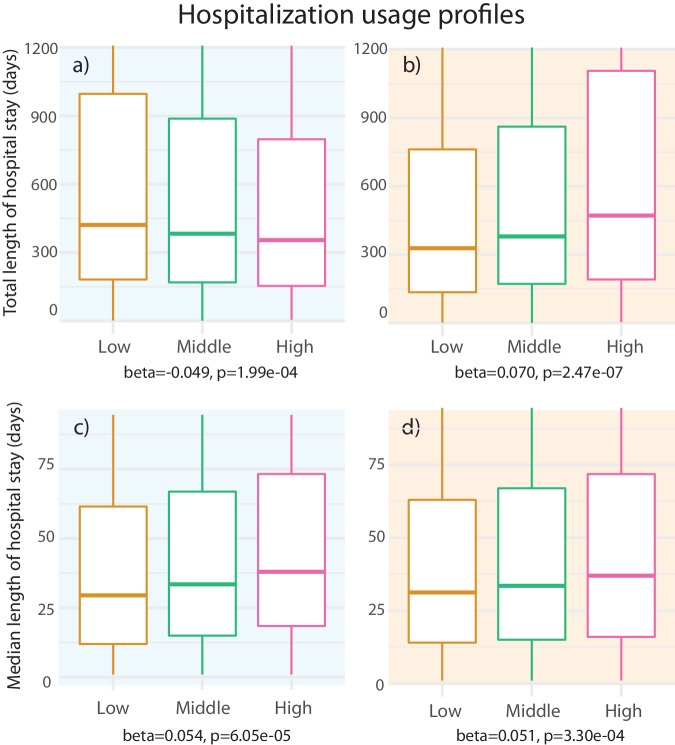
Hospital usage profiles associated with SZ-PGS and EA-PGS. The upper panels (**a**, **b**) display the relationship between the EA-PGS and the SZ-PGS and the total length of stay for the first 15 years post psychotic illness onset. The two lower panels (**c** + **d**) show the relationship between the EA-PGS and the SZ-PGS and the median length of each separate hospital visit for the same time-period. The EA-PGS and the SZ-PGS associated with two distinctly different hospitalization profiles. A low EA-PGS associates with a longer total time spent at hospital, but the total time was composed of shorter, more frequent visits, while a high SZ-PRS associated with longer total time spent at hospital and longer individual hospital stays. [PGS strata: Low = <20% (*n* = 1096); Middle = 20–60% (*n* = 3287); High = >80% (*n* = 1096); total *n* = 5479].

The apparent independent effects of SZ-PGS and EA-PGS on the psychiatric hospitalization burden let us to characterize the patterns of hospital admissions more closely. The analysis revealed that the SZ-PGS and the EA-PGS were associated with distinct profiles of hospital usage [Fig. 4]. While both a high SZ-PGS and a low EA-PGS were associated with a longer total time spent in hospital (*β* = 0.070, *p* = 2.47e-07, *β* = 0.049, *p* = 1.99e-04 respectively), the composition of the hospital stays was different. A high SZ-PGS associated with longer individual stays in the hospital (*β* = 0.051, *p* = 3.30e-04). A low EA-PGS, in contrast, associated with shorter (*β* = −0.054, *p* = 6.05e-05), but more frequent, hospital stays. Taken together, the hospital usage profile of individuals with a high SZ-PGS showed a pattern more likely to reflect a disorder with increased disease-severity and greater need of medical care. On the other hand, the hospital usage profile associated with a low EA-PGS resembled a pattern often called a “revolving door” phenomenon, characterized by short and frequent visits, regularly observed when drug and/or substance abuse is present [29, 30].

### Substance use disorder partly mediates the EA PGS’s effect on psychiatric hospitalization burden

We next wondered about the potential reasons for the observed differences in the patterns of hospital stays associated with SZ-PGS and EA-PGS. Because substance use disorder (SUD) is known to be related to a revolving door pattern, and is a common comorbidity in psychotic disorders [31], we examined whether the EA-PGS’s hospitalization pattern also was related to SUD in our study. For this purpose, we studied the association of SUD in individuals with schizophrenia (*n* = 1763, “Methods”) to the pattern of hospital treatment periods. In line with previous reports [32–35], a diagnosis of SUD had a major impact on the psychiatric hospitalization burden (*β* = 0.50, *p* = 2.95e-65). Interestingly, the association between the SUD-endpoint and the EA-PGS was far stronger (OR = 0.68 [0.64–0.72], p = 1.37e-35) than for SZ-PGS (OR = 0.99 [0.94–1.056) or the other assessed PGSs [Fig S5]. The EA-PGS also displayed a strong association with self-reported cannabis usage that was measured at study inclusion, while the SZ-PGS was not associated with either the SUD-endpoint (*p* = 0.85) or self-reported cannabis usage (*p* = 0.52) [Fig S6]. Importantly, we observed that the EA-PGS displayed the same effects in a non-psychiatric population (*n* = 30,544) in the Finnish nation-wide FinnGen study, where SUD were consistently associated to the EA-PGS but not with the SZ-PGS [Supplementary appendix and Supplementary methods]. Further, we observed that outcomes that were primarily associated with the EA-PGS tended to share the EA-PGS’s hospitalization usage profile, and vice versa for the SZ-PGS [Fig S7]. The revolving door pattern was most evident for individuals with SUD, who had a much shorter median length of stay (21 vs 41 days, *p* = 4.16e-64) while still having a longer total time spent in the hospital (*β* = 0.22, *p* = 6.65e-15). The SUD-endpoint showed a time-dependent effect on psychiatric hospitalizations, suggesting it might act as a direct effector on the need for psychiatric hospital care, and not just a marker for a poor outcome [Fig S8].

The intimate relationship between the SUD-endpoint and the EA-PGS lead us to hypothesize that the SUD-endpoint could act as a mediator for the EA-PGS’s effect on psychiatric hospitalizations. This hypothesis was further supported by a complete attenuation of the association between the EA-PGS and psychiatric hospitalization burden when all individuals with the SUD-endpoint were excluded from the analysis (*β* = −0.020, *p* = 0.23, after SUD exclusion) [Fig S9]. We used structural equation modeling (SEM) to assess potential causal directions between the EA-PGS, SUD-endpoint and psychiatric hospitalizations. The SEM analysis gave strong support that the EA PGS’s effect on psychiatric hospitalization burden was, indeed, partly mediated via its effect on the SUD-endpoint (*β* = −0.040, *p* = 1.03e-24) [Fig S10]. In the mediation model the highest acquired educational level for each individual was also included as a possible second mediator, given its apparent and immediate relationship with the EA-PGS. However, unlike the SUD-endpoint, the highest acquired educational level did not display a noticeable temporal effect on psychiatric hospitalization burden [Fig S11]. Excluding the acquired educational level from the model marginally strengthened the EA-PGS’s indirect mediation effect via the SUD-endpoint (*β* = −0.044, *p* = 1.66e-27).

## Discussion

We investigated the genetic contribution to diagnostic progression and disease severity within the spectrum of psychotic diagnoses in the SUPER-Finland study, which builds on nation-wide, full coverage, high-quality medical healthcare registries in Finland. While PGSs for psychiatric disorders are still insufficient for screening purposes in the general population [2, 36] their use for prognostic prediction would meet a great clinical need for patients with a psychotic illness. Hitherto, only a few clinical predictors have been associated with a poor outcome following first psychotic episode [37]. Here, using this unique dataset, with decades of medical follow-up, we were able to characterize the disease-course development in an innovative way by longitudinally tracking past disease-course severity and investigating the polygenetic architecture contributing to disease outcome. High SZ-PGS was associated with progression from a lower ranked psychotic disorder to schizophrenia and a greater need for psychiatric hospital care in individuals with schizophrenia. The EA-PGS had the largest impact on psychiatric hospitalization burden. The effect was in part explained by its influence on the risk of acquiring SUD, and it was independent from the effect of SZ-PGS. So, although both a high SZ-PGS and a low EA-PGS increased the total psychiatric hospitalization burden over time, the effects were due to different reasons. This conclusion was also supported by evidence that the EA-PGS displayed the same effects in a non-psychiatric population. High SZ-PGS was associated with a hospital usage profile more likely to reflect severe psychotic disease, while low EA-PGS was associated with a hospitalization pattern regularly observed when SUD is present [29, 30]. These results are in support of a future potential utility for polygenic scores to help clinicians in prognostic guidance and to help in planning supportive efforts, especially focusing on SUD.

### Diagnostic progression of psychotic disorders

The degree and quality of psychotic symptoms can progress over the disease-course and the diagnosis is therefore often adjusted over time [4]. Our findings suggest, however, that the individuals who later will progress to schizophrenia from an initial lower ranked psychotic disorder have a similar need for psychiatric hospital care as other individuals with schizophrenia already from the start of their psychotic illness. This was in stark contrast to the significantly lower need for hospital care observed for individuals who remained at a lower ranked diagnosis. The group that progressed to schizophrenia had their psychotic illness onset at the same age as other individuals with schizophrenia but received their schizophrenia diagnosis substantially later (diagnosis delay was almost 6 years). Although these results could be affected by changing diagnostic practices over time, the schizophrenia diagnosis delay for the progression group was seen across all studied time periods (Fig SA7). The individuals who progressed to schizophrenia also had higher SZ-PGS than individuals who remained at lower ranked psychotic diagnoses, together suggesting that initial diagnostic difficulties might partially explain these findings or at least that their disease-course development was largely determined already at the start of first psychotic illness onset. Interestingly a strong sex imbalance (*p* = 1.08e-13) was seen between the progression group (53.1% females/46.9% males), and the group that received schizophrenia as the first major psychotic disorder (40.8% females/59.2% males). This observation could potentially, in part, explain why many studies have found women to be older at the onset of schizophrenia compared to males [38, 39]. However, the progression group had a slightly higher MDD-PGS than other individuals with schizophrenia, signifying that their symptomatology could have differed and possibly involved more affective symptoms.

### Psychiatric hospitalization burden in schizophrenia

Recent studies in hypertension and age-related macular degeneration have shown that common genetic variants can add prognostic value, beyond their ability to cross-sectionally predict the trait [40, 41]. In schizophrenia, we showed that individuals with high SZ-PGS had an increased psychiatric hospitalization burden, and that they displayed a hospitalization usage profile indicative of a more severe disease. The results suggest that the genetic factors that are important for the development of schizophrenia also influence the disease-course. However, the schizophrenia GWAS used to construct the PGS [2] were likely enriched for severe cases and included the CLOZUK cohort with more than 5000 treatment resistant cases (proxied by clozapine usage). Treatment resistance schizophrenia have recently been demonstrated to be a trait with independent heritability, and that a high clozapine dose associates with high SZ-PGS [42, 43]. However, the genetic architecture for treatment resistant schizophrenia cases has been shown to be very similar (r_g_=0.954) to non-treatment resistant cases, suggesting that a bias would only be moderate [44]. Further, treatment resistance is difficult to measure in registry-based research [45], and although a prescription of clozapine is often used as a proxy for treatment resistance, there is wide geographical variation in clozapine use suggesting that not all individuals who would benefit from clozapine treatment actually receive it [46]. In a sensitivity analysis we also showed that the association between the SZ-PGS and increased need for psychiatric hospitalizations remained in non-clozapine users.

### The EA-PGS had the largest influence on psychiatric hospitalization burden

A high EA-PGS is known to associate with positive psychosocial outcomes, both within psychiatric and somatic disorders [23, 47], and in our study the EA-PGS had the largest influence on psychiatric hospitalization burden. The EA-PGS, unlike the SZ-PGS, was associated with frequent short visits in resemblance with the SUD-related revolving door phenomenon. Previously, low performance in primary school has been causally linked with future drug abuse [48]. In line with these observations, we observed that the SUD-endpoint, which had a major impact on the hospitalization burden, predominantly associated with the EA-PGS, but not with the SZ-PGS (*p* = 0.85). Also, after excluding all individuals with a SUD diagnosis (*n* = 1763), the EA-PGS were no longer associated with hospital burden (*p* = 0.23, Fig S9). The SEM analysis showed a strong indirect effect of the EA-PGS via the SUD-endpoint and together the results form credible support that the association between a low EA-PGS and increased psychiatric hospitalization burden was mediated in part by SUD. That a low EA-PGS was associated with an unfavorable outcome via SUD, independent of the SZ-PGS, indirectly suggest that initiatives focusing on treatment and/or prevention of SUD could prevent frequent hospitalizations and improve the outcome of people with dual diagnoses. We draw this conclusion because our results do not support SUD to be a consequence of a more severe psychotic disease, but instead that SUD leads to a poor hospitalization outcome independently of an individual’s genetic liability to schizophrenia.

### Study limitations

Although this study has several strengths, foremost due to the use of nation-wide, full coverage, medial healthcare registry data in a sizable cohort, there are some important limitations. The study includes longitudinal health-care register data over a multi-generational cohort of individuals (born between 1927–2000) that have been subject to changing clinical practices over the last 50 years. In addition, there are inherent stratification biases due to the recruitment. First, because we require an occurrence for a psychotic disorder, or even a diagnosis of schizophrenia, we are at risk for index event bias. However, when focusing on hospitalization outcomes, we confided the analysis to schizophrenia cases to make the results interpretable. Further, all study participants needed to be alive at study inclusion (2016–2018) and capable to consenting, resulting in potential underrepresentation of older individuals, while all young study participants needed to already have received a psychotic diagnosis to be included. This may impact comparisons across generations because of different time-constrains in their individual disease course-development. Our hospitalization metric aimed to mitigate these generational differences, but this issue can never fully be overcome.

## Summary

In summary, we show that individuals with high SZ-PGS were more likely to progress from another major psychotic diagnosis to schizophrenia and had an increased need for psychiatric hospital care. Individuals with low EA-PGS also had an increased need for psychiatric hospital care, but not for the same reasons as individuals with a high SZ-PGS. Instead, we find support that the association between a low EA-PGS and increased psychiatric hospitalizations was partly mediated by substance use disorder, resulting in a specific disease-trajectory that potentially could be targeted with preventive efforts.

## Supplementary information

Supplementary methods

Supplementary appendix

Supplementary Figures

## Data Availability

This study includes individual genotypes and sensitive health care data. We are not allowed to transfer the data outside the secure environments at the Institute for Molecular Medicine Finland, Helsinki, Finland and The Stanley Center, Broad Institute of MIT and Harvard, Cambrigde, USA. However, data access can be managed in collaboration with the authors. For data access requests, contact OP.
