# Supplementary material for: Genetic contribution to disease-course severity and progression in the SUPER-Finland study, a cohort of 10,403 individuals with psychotic disorders
Source: Mol Psychiatry. 2024 Apr 1;29(9):2733–41. doi: 10.1038/s41380-024-02516-6 (PMC11420086; doi:10.1038/s41380-024-02516-6)
Supplement: Supplementary file 1 — Supplementary methods [file 41380_2024_2516_MOESM1_ESM.doc]

**Supplementary methods**

**INDEX**

The SUPER-Finland study ………………………………………… 2

The FinnGen Study ………………………………………… 3

Quality control and imputation of genotypes ………………………………………… 4

Cross-sectional endpoint definitions ………………………………………… 6-7

Clinical (registry-based) endpoint definitions ………………………………………… 8-9

Discrepancies between self-reported and registry-based diagnoses …………………….. 10-11

Polygenic score construction and summary statistics ………………………………………… 12

Statistical models used ………………………………………… 13-14

References ………………………………………… 15

**The SUPER-Finland study­­­­­**

The SUPER-Finland study is part of the Stanley Global Neuropsychiatric Genetics initiative (<https://www.broadinstitute.org/stanley-center-psychiatric-research/stanley-global>) and includes 10,403 individuals, with active consent, and at least one episode of psychotic episode. The study strategy aimed to recruit individuals from the full psychotic spectrum and comprehensively collect cases with schizophrenia spectrum psychotic disorder (ICD-10 codes F20, F22-29), bipolar I disorder with psychosis (F30.2, F31.2, F31.5), and major depressive disorder with psychotic features (F32.3 and F33.3) from in- and outpatient psychiatric and general care units. The recruitment was performed by psychiatrist and psychiatric nurses that were part of the study staff. The recruitment process was nation-wide and involved all hospital districts in Finland with the intent to collect a representative sample of psychotic disorders in Finland [**Fig SM1**].

**Fig SM1.** Geographical distribution of the SUPER participants. Each blue dot represents an individual study participant. The 7 largest urban areas in Finland are marked.

At inclusion, the study participants underwent a protocol guided interview that was conducted in person by study personnel. This included a comprehensive questionnaire and cognitive testing using the Cambridge Neuropsychological Test Automated Battery (CANTAB). Current medication and dosages were also collected at inclusion. The number of individuals who declined participation in the study is unknown.

The study description can be found at:

<https://thl.fi/en/web/thl-biobank/for-researchers/sample-collections/super-study>


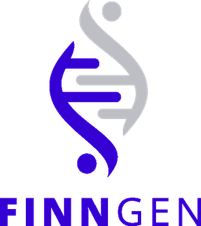
**The FinnGen study** (validation analysis only)

The FinnGen study includes genetic data from 377 277 individuals (data freeze 9) from the Finnish population (<https://www.finngen.fi/en>). FinnGen is a national Finnish initiative that during 2023 will have collected and genotyped approximately 10% av the Finnish population (1). It is a public-private partnership where 13 international pharmaceutical companies collaborate with Finnish universities, university hospitals and nine national biobanks. The full coverage and nation-wide Finnish medical health care registries have been linked to each participant in FinnGen, providing a unique biobank resource. The overall study protocol was approved by the Coordinating Ethics Committee of the Hospital District of Helsinki and Uusimaa (HUS) (number HUS/990/2017). The validation analysis was performed in a non-psychiatric population in the two population-based cohorts FINRISK (2) and H2000 (3) (n=30 544), included in FinnGen. The validation analysis be found the **Supplementary appendix.**

**Quality control and imputation of genotypes**

**Fig SM2. Flowchart of genotype processing**

The Illumina Global Screening Array chip (version 1) was used for genotyping. After genotypic quality control the study included 8 354 individuals that also had at least one of the 4 major psychotic diagnoses recorded in the registries [**Table** **SM1**]. Diagnoses recorded in the interview at study inclusion were not considered. Samples were lost when sent to genotyping (10 008 samples were successfully genotyped), removed because of bad genotyping quality (n=111) and non-European ancestry (n=71). [**Fig SM3**].

**Table SM1.**

| Individuals with active consent. | Passed genotype QC filters. | Any psychosis-related event recorded in the registers. | Non-ranked psychotic diagnoses as most severe diagnosis. | A registry recorded diagnosis of any of the four major psychotic disorders (psychotic MDD, BD, SAD, SZ). |
| --- | --- | --- | --- | --- |
| n=10 403 | n=9 826 | n=9 421 | n=1 067 | n=8 354 |

**Fig SM3.** Samples form 5 different ancestries (1000 Genomes, n=2 504) compared to the study participants (black). In total the study samples contained 71 ancestral outliers (>5 SD from EUR samples, marked by black box). [EAS = East Asian, AMR = Ad Mixed American, SAS = South Asian, AFR = African, EUR = European, FIN = Finnish, SUPER = SUPER study participants.]. Computations were performed using the LASER suit (4).

**Cross-sectional endpoint definitions**

**Cross-sectional data collected at study inclusion used in the analyses:**

*(Only analyzed in individuals with schizophrenia, n=5 479)*

*Highest acquired educational level:* The highest acquired educational level was defined according to the Finnish Health 2000 general population survey (5) into three levels: “Basic/low” education, “secondary/middle” education and “higher” education. Basic education was defined as *not* having completed vocational school, secondary education was defined as having completed vocational school and passed the matriculation exam (age 19) and higher education was defined as having a degree from a higher educational institution such as a university or similar (median age of receiving a degree for higher education in Finland was 28 years in 2010). The educational level was coded as 0 (n=2 181),1 (n=2 404) and 2 (n=838), corresponding to the three educational levels, respectively. The education information was completely missing for 56 individuals. The information regarding the highest acquired educational level was recorded as part of the interview at study inclusion. For 53 individuals the educational level was missing from the interview and information from the School Registry was used.

*Living situation:* The participants were asked about their living situation during the interview and the answers were documented in open answers. Supported living (n=1 881) was defined as all answers that specified that external support was received. Living with parents was not considered as having a supported living. Living situation was recorded and could unequivocally be placed for one of the above categories for 4 970 individuals out of 5 479.

*Suicide attempts:* The participants were asked if they ever had attempted to commit suicide. Three levels of answers were considered: “Never”, “Once”, and “More than once”, which were codes as 0 (n=3 177), 1 (n=1 050) and 2 (n=1 093) respectively. 159 individuals had missing information. Suicide attempts were recorded for 5 320 individuals out of 5 479.

*Cannabis usage:* The participants was asked whether they use cannabis and how often they use it. The answers were grouped into three categories: “Never”, “Once in a while” and “Often (>more than 50 times)”. The cannabis usage frequency was then coded as 0

(n=3 880), 1 (n=851) and 2 (n=596), respectively. 152 individuals had missing information.

*Clozapine use:* Current use of clozapine was used as a proxy for treatment resistant schizophrenia. At study inclusion the participants were interviewed about their current medications and all individuals with a current use of clozapine (all dosages) were coded as clozapine users (n=2 102). Everyone else was considered not to have used clozapine

(n=3 377). Previous use of clozapine was not recorded in the study.

*Occupation:* The information about the participants current occupation was recorded at study inclusion and occupational status was coded as “No current job” (n=4 841) or “Current job” (n=396), missing data (n=242). Students were considered to have an occupation.

*Repeat school year:* All participants were asked at study inclusion if they had needed to repeat a grade in school. Coded as “No” (n=1 989) and “Yes” (n=736), missing data

(n=2 754).

*Functioning at discharge:* Since 1992, all psychiatric wards in Finland rate their patients general functioning level at discharge using the Global Assessment Scale (GAS) (6) at discharge. The patients mean GAS score (average over all hospital visits) was used as a quantitative outcome. Out of the 5 479 individuals, 5 116 had at least one hospital visit where their function at discharge had been rated using GAS.

*Adjusted Paired Associates Learning (PAL) test:* At study inclusion the participants underwent the Cambridge Neuropsychological Test Automated Battery (CANTAB) test. Adjusted total PAL errors was chosen as the parameter to best represent cognition. Only test scores that had been rated as “complete and reliable” by the supervising research personnel were used (n=3 411). The test score was transformed using a rank-based inverse normal transformation to account for a bi-modal distribution. It is important to note that the strict requirement for test completion likely biases the analysis towards overall better-functioning individuals.

**Clinical (registry-based) endpoint definitions**

All clinical endpoints used in the study were obtained from ICD codes recorded in the Finnish National Care Register for Health Care and Register of Primary Health Care Visits. A complete description of the Finnish Health Care Registers can be found here: Finnish National Care Register for Health Care: <https://thl.fi/en/web/thlfi-en/statistics-and-data/data-and-services/register-descriptions/care-register-for-health-care>; Register of Primary Health Care Visits: <https://thl.fi/en/web/thlfi-en/statistics-and-data/data-and-services/register-descriptions/register-of-primary-health-care-visits>.

It is important to **NOTE** that the meaning of the ICD codes in the Finnish ICD system are not always equivalent to the codes used in the international ICD system (WHO) or det clinically modified ICD system used in the U.S (ICD-CM). Finnish ICD-10 codes can be found here: <https://www.julkari.fi/handle/10024/80324>).

**Clinical endpoints obtained from the medical health registries**

*Major psychotic diagnoses*: The study focused on the four major psychotic diagnoses (SZ, SAD, BD, MDD) defined by the occurrence of the specific ICD codes in the medical registries (**Table SM2**). Only diagnoses recorded in the health care registries were considered.

**Table SM2.**

| Diagnosis | ICD 10 | ICD 9 | ICD 8 |
| --- | --- | --- | --- |
| Schizophrenia (SZ) | F20 | 295[0-3]|295[5-6]|295[8-9] | 295[0-6]|295[8-9] |
| Schizoaffective disorder (SAD) | F25 | 2957 | 2957 |
| Bipolar disorder (BD) | F30.2|F31 | 2962|2963|2964|2967 | 2961|2962|2963|2968 |
| Psychotic MDD | F32.3|F33.3 | 2961E | NA |

*Age of psychotic illness onset:* Because all the studied individuals (n=8 354) already had at least one of the four major psychotic disorders, we used a broad definition of the age of psychotic illness onset, with the intent to capture the earliest stage of the disease-course. All recorded affective, non-affective, substance-induced psychotic disorders and psychotic disorders caused by a general medical condition were included by searching the register using the following search string (ICD10/9/8): “^F2[0-9]|^295[0-9][A-Z]|^29[8][0-9][A-Z]|^295[0-9][0-9]|^29[7-9][0-9][0-9]|^F30$|^F30[2-9]|^F31|^F3[2-3]3|^2960[A-Z]|^296[2-7][A-Z]|^296[1-9][0-9]|^F1007|^F1[0-9]0[3-4]|^F1[0-9][5-9]|^291[0-4][A-Z]|^292[1-9][A-Z]|^291[0-9][0-9]|^F0[4-5]|^F06[0-2]|F068|^F079|^F09|^293[0-9][A-C]|^29[2-3][0-9][0-9]|^294[0-2|4-9][0-9]|^F00|^F01|^F02|^F03|^290|^2941A|^29430|^2961E|^F531”

*Substance use disorder (SUD) endpoint:* Substance use disorder (SUD) was defined as any-time diagnosis indicating a substance abuse of substance misuse [ICD10 equivalents F10-F16, F18-F19, n=1 763]. Tobacco/nicotine use (F17) were not considered a substance misuse and excluded from the SUD definition.

*Age of onset:* The age of onset for all clinical endpoints was set to the date of the first registry recorded ICD diagnosis included in the endpoint. The year and month of birth was available for all study participants. The birthdate was set to the 15^th^ of the birth month for each participant.

**Discrepancies between self-reported and registry-based diagnoses.**

In total 405 individuals did not have a psychotic disorder diagnosis recorded in the registry. All participating individuals had, in their own view, had a psychotic disorder or episode. Lack of register diagnosis could be due to several factors. Firstly, although uncommon, they could have attended private outpatient care which is not included in the registers. Because 314 of these individuals had a self-reported psychotic diagnosis, and >80% also had other psychiatric diagnosis in the registers. Short psychotic episodes could have occurred e.g. in patients with borderline personality disorder without a specific diagnosis being formally set. We also know from our previous studies that there are individuals with psychotic disorders who have had very limited or no treatment contacts during their lifetime (7). In addition, study personnel reported that sometimes during and/or after the interview process it became apparent that a study individual, although having psychiatric problems, did not necessarily have a psychotic disorder. However, because the questionnaire and interview procedures often were quite exhausting for the study individuals, study personnel reported that it felt wrong to exclude the patient on the spot, and instead these individuals were sometimes included. We do also observe that the individuals without a registry recorded psychotic diagnosis had a different genetic architecture compared to the rest of the cohort (**Fig SM4,** next page), in support of our strategy to only consider registry recorded diagnoses.


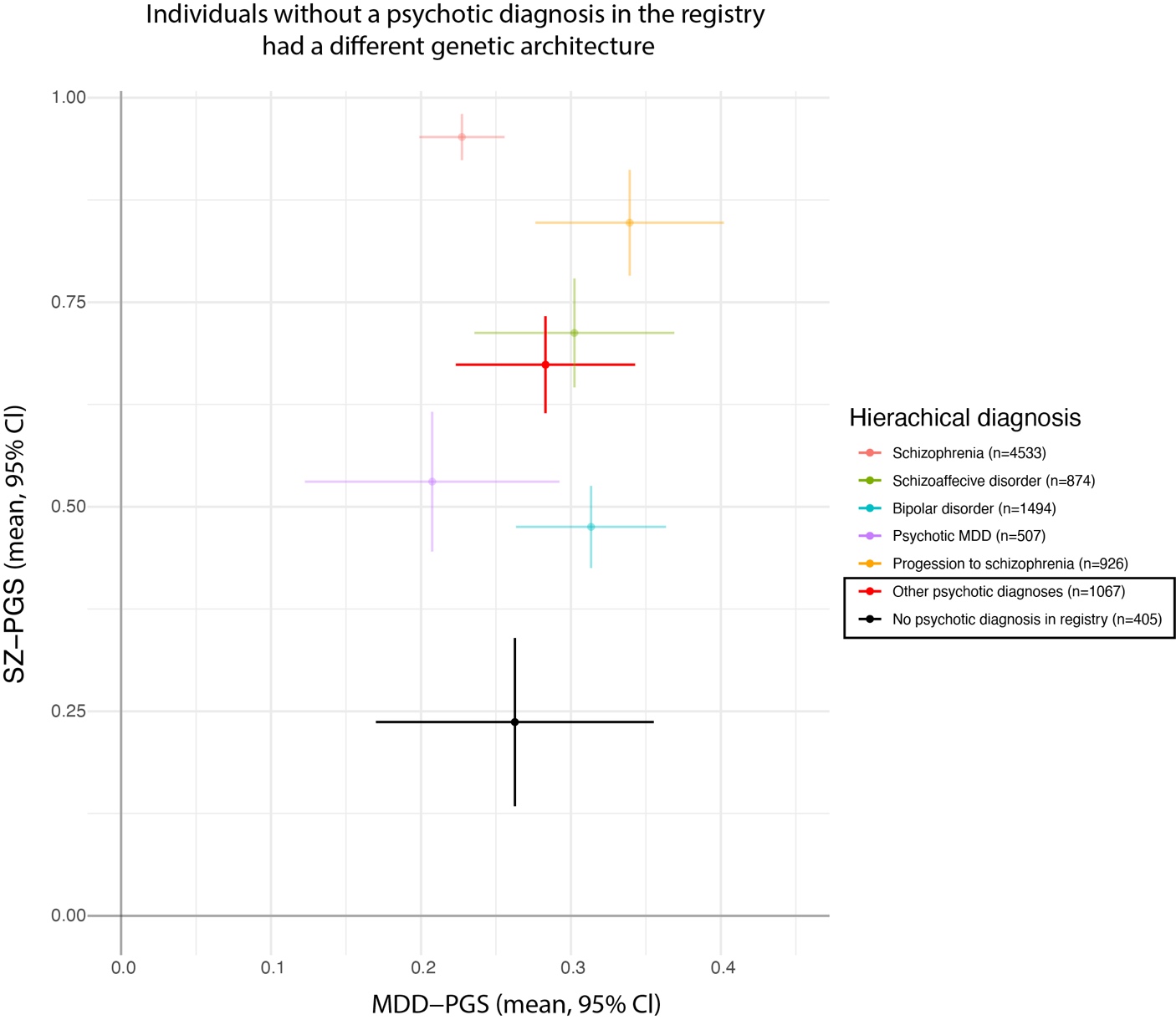


**Fig SM4.** The plot displays the mean SZ-PGS on y-axis and the mean MDD-PGS on the x-axis. The plot reproduces the **Fig1b** in the main manuscript, but here also the two groups not considered in the current study are included (i.e ‘Other psychotic diagnoses’ and ‘No psychotic diagnosis in registry’). The four major psychotic disorders are plotted in the background for reference (faint colors). The individuals with other psychotic diagnoses, although likely a very heterogenous group, had on average a similar genetic architecture as the rest of the SUPER-Finland individuals, while the individuals with no psychotic diagnosis in the registry had a substantially lower SZ-PGS. [Error bars display 95% Cl of the mean]

**Polygenic score construction**

Polygenic scores (PGSs), were constructed using MegaPRS (8) and PRS-CS (only used in the FinnGen sub-analysis) (9). The software perform well for psychiatric traits compared to other advanced PGS methods (10).

The MegaPRS software was run using standard settings recommended by the authors (<https://dougspeed.com/megaprs/>) using the BLD-LDAK heritability model and BayesR-SS to construct the prediction model. Model parameters were selected using pseudo cross-validation. The 1000 genomes non-Finnish European subpopulation was used as reference to estimate SNP-SNP correlations. Only SNPs with a minor allele frequency ≥1% and an imputation score ≥0.95 (when available) was included for PGS construction. The polygenic scores in FinnGen were pre-calculated with PRS-CS by the FinnGen Analysis Team according to the FinnGen pipeline (<https://github.com/FINNGEN/CS-PRS-pipeline>). The summary statistics used for the PGS construction are listed in **Table SM3** (below). Note, that for the FinnGen analysis [**Supplementary appendix**], the EA-PGS was constructed from Lee at al (2018) (11) instead of Okbay et al (12). The two summary statistics for educational attainment included the same samples, but the recorded UKBB educational level had been remapped in the more recent Okbay et al (2022).

**Table SM3. Summary statistics used for polygenic score construction.**

| **Trait** | **Study** | **Study type** | **N participants** | **Link to study:** | **Ancestry** |
| --- | --- | --- | --- | --- | --- |
| Schizophrenia | Trubetskoy et al. Nature (2022) (13) | meta-analysis | 67,390 (cases)  94,015 (controls) | [Link](https://www.nature.com/articles/s41586-022-04434-5) | Core GWAS: European (80%); East asian (20%) |
| Bipolar disorder | Mullins et al. Nat Genet (2021) (14) | meta-analysis | 41,917 (cases)  371,549 (controls) | [Link](https://www.nature.com/articles/s41588-021-00857-4) | European |
| Major depressive disorder | Wray et al. Nat Genet (2018) (15) | meta-analysis | 59,851 (cases)  113,154 (controls) [Excluding 23andMe] | [Link](https://www.nature.com/articles/s41588-018-0090-3) | European |
| Educational attainment | Okbay et al Nat Genet (2022) (12) | meta-analysis | 765,283  [Excluding 23andMe] | [Link](https://www.nature.com/articles/s41588-022-01016-z) | European |
| Intelligence | Savage et al. Nat Genet (2018) (16) | meta-analysis | 269,867 | [Link](https://www.nature.com/articles/s41588-018-0152-6) | European |
| Cannabis use disorder | Johnson et al LANCET PSYCHIAT (2020) (17) | meta-analysis | 13,990 (cases)  343,002 (controls) | [Link](https://www.thelancet.com/journals/lanpsy/article/PIIS2215-0366(20)30339-4/fulltext) | European |
| Alcohol dependence | Walters et al. Nat Neurosci (2018) (18) | meta-analysis | 11,569 (cases)  34,999 (controls) | [Link](https://www.nature.com/articles/s41593-018-0275-1) | European |

The SUPER-Finland study was not part of any of the studies used for polygenic score construction.

**Statistical models used**

*Cox regression model*

Using a cox proportional hazard model, we assessed the SZ-PGS hazard ratio for converting from an initial lower ranked psychotic disorder (psychotic MDD, BD or SAD) to schizophrenia [**Fig 1a, main manuscript**]. The zero time-point was set to the time-point when the first diagnosis of each lower ranked psychotic disorder (psychotic MDD, BD or SAD) was recorded. Individuals were censored due to death or time-point of end of follow-up. Time to event was measured as time (days) to schizophrenia diagnosis. Hazard ratios and p-values was calculated using the SZ-PRS as a continuous variable and the displayed SZ-PGS strata (‘Low’, ‘Middle’, ‘High’) was only for graphical purposes. Sex, year of birth and the 10 first principal components were used as covariates. Because of the structure in the hierarchical diagnostic system used, a single individual could overlap more than one progression category. As an example, if an individual first received a diagnosis of psychotic MDD (the lowest ranked diagnosis) and later progressed to schizophrenia, that individual was placed in the category that progressed form psychotic MDD to SZ. However, if the same individual had received a BD diagnosis after receiving the psychotic MDD diagnosis, but prior to the SZ diagnosis, that person was also placed in the category that progressed from BD to SZ, because at some point in time that individual had BD as his/her highest ranked psychotic disorder. Note that the hierarchy is unidirectional, meaning that an individual cannot progress from a higher ranked diagnosis to a lower ranked diagnosis. In total 176 individuals were placed in more than one progression category. In the overall analysis, the individuals were only counted once (total n=926).

*Linear and logistic regression models*

Dichotomous outcome variables were assessed using logistic regression models, while continuous variables were assessed using linear regression models. Transformations were applied when appropriate. Poisson regression models were used when the outcomes had a distinct count data distribution (e.g educational level with counts 0,1 and 2). Sex, year of birth and the 10 first principal components were used as covariates in all models.

*Hospitalization usage profiles*

To estimate the hospitalization usage profiles the median length of each individual’s psychiatric hospital visits, as well as the total hospital length of stay, was calculated for the first 15 years of the disease-course (counted from the time-point of the first record of psychotic illness). We focused on the first 15 years of the disease course because we wanted the majority of the individuals to have complete follow-up time (71% had at least 15 years of follow-up since illness onset). Further, we wanted the measurement to be comparable between individuals and not to be biased by the cohorts age distribution, because the older individuals had a much longer total (retrospective) follow-up time.

*Psychiatric hospitalization burden metric*

Hospitalization burden can be considered a valid measurement for disease severity in schizophrenia (19, 20), but can be calculated in many forms. We calculated the qualitative need for psychiatric in-patient care (0/No or 1/Yes) for each individual and year, defined as being hospitalized at least one night primary due to a psychiatric diagnosis. This was an effort to mitigate the dramatic decline in the number of psychiatric hospital beds in Finland during the last 50 years (21), and to take the yearly hospital usage distribution into account [details in **Supplemental appendix**]. We believe this approach will best reflect an individual’s psychiatric hospitalization burden over time and facilitate comparisons across generations. For the analyses, the psychiatric hospitalization burden was used as an outcome in a linear model, where the individuals’ average yearly need for psychiatric hospital care was calculated. The average yearly need for psychiatric hospital care was calculated for the first 15 years of the disease-course (counted from the time-point of the first record of psychotic illness). The time-frame chosen aimed to best facilitate comparisons between individuals and across generations for the reasons listed above. Prior to illness onset the yearly need for psychiatric hospital care was on average very low, which was why this time-period was not assessed. To test the robustness of the standard linear model we also used a linear mixed model where the psychiatric hospitalization status each year (the outcome) were treated as a repeated measurement with sample-ID and time-of-measurement as random variables. The results showed in principle mirroring results [**Supplemental appendix**], which was why we chose to keep the strategy of using a standard linear model. For **Fig3a** (main manuscript), the effect size and p-value were calculated using the individuals´ average yearly need for psychiatric hospital care between the age of 20-50 years. However, we believed that aligning the hospitalization data for the time-point of disease-course onset (first recorded sign of psychotic illness) was a more valid approach that better allowed for comparisons between individuals and across generations. See **Supplemental appendix** for full details.

*Structural equation modelling analysis – mediation model*

The R package lavaan (22) was used for the structural equation modelling analysis. The mediation model included substance use disorder (SUD) and the highest acquired educational level as the two potential mediators. The highest acquired educational level was treated as a pseudo continuous variable (0,1,2) and the SUD variable was converted to a pseudo continuous variable (0=no SUD, 1= age of diagnosis > median age, 2= age of diagnosis < median age). The average yearly psychiatric hospitalization burden for the first 15 years of psychotic illness was used as outcome. Sex, year of birth and the 10 first principal components were used as covariates. The model used was a just-identified model with 0 degrees of freedom. Standard errors were calculated using bootstrapping (5,000 iterations) to produce more robust estimates.

**References**
