# Supplementary material for: Genetic contribution to disease-course severity and progression in the SUPER-Finland study, a cohort of 10,403 individuals with psychotic disorders
Source: Mol Psychiatry. 2024 Apr 1;29(9):2733–41. doi: 10.1038/s41380-024-02516-6 (PMC11420086; doi:10.1038/s41380-024-02516-6)
Supplement: Supplementary file 2 — Supplementary appendix [file 41380_2024_2516_MOESM2_ESM.doc]

**Supplementary appendix**

**INDEX**

Construction of the psychiatric hospitalization

burden metric ………………………………………… 2-6

Diagnostic progression towards schizophrenia ………………………………………… 8-10

Substance use disorder (SUD) in a non-psychiatric

population in FinnGen. ………………………………………… 11-12

References ………………………………………… 13

**Psychiatric hospitalization burden**

When constructing the hospitalization metric, we decided to focus only on psychiatric hospital admissions, because within the SUPER-Finland study, hospital admissions primarily due to psychiatric diagnoses were much more common than hospital admissions primarily due to non-psychiatric diagnoses [**Fig SA1**]. Non-psychiatric causes for hospital care were also relatively more common in older, as well as very young, individuals. Further, they are inherently more difficult to compare between individuals because of the wide range of diagnoses from different organ systems.

**Fig SA1.** For individuals with schizophrenia, hospital admissions primarily due to psychiatric diagnoses were more common and likely represents a better measurement of psychotic disease severity than all-cause hospitalizations, which also had a different age-profile.

**Construction of the psychiatric hospital burden metric**

We observed that the correlation between the number of hospital admissions and the total length of hospital stay was low (r=0.29 / r^2^=0.08). Many individuals were frequently admitted, but discharged quickly, while some have fewer but longer hospital stays. Thus, it was difficult to compare individuals with many short hospital admissions with individuals who mainly had long, but fewer hospital stays for the same time-period. We also observed that for any given year the most likely status of an individual with schizophrenia was to have had 0 psychiatric hospital admissions during that specific year. The peak hospitalization burden for individuals with schizophrenia was reach at age 30, nonetheless the most likely event for a single individual at age 30 was still to have had 0 hospital admissions during that year (63% had 0 hospital admissions at age 30). As seen in **Fig SA2a**, the noticeable difference was not weather an individual had 10, 100 or 200 hospital days for a given year, but whether an individual had been hospitalized primarily due to a psychiatric diagnosis or not during that year. **Fig SA2b** also shows the decline of psychiatric hospital care in Finland between 1996-2022. From 1996 the number of psychiatric in-hospital patients per 1000 inhabitants has decreased less than psychiatric care days per 1000 inhabitants. This is inline with a recent meta-analysis showing that hospital stays for psychotic episodes have become shorter over time, but re-admissions and the proportion of people needing psychiatric hospital care have not (1). It is important to note that in 1996 the number of hospital bed had already decreased in Finland, from its peak in the 1970 with 4 psychiatric beds per 1000 individuals to the current 0.6 per 1000 individuals (2).

­­­­

**Fig SA2.** **a)** Individuals with schizophrenia had, on average, their hospitalization peak at age 30. However, the most common state of an individual at age 30 was to not have been hospitalized for a primary psychiatric diagnosis during that year. **b)** Data Finnish Institute for Health and Welfare show that the proportion (nr per 1000 inhabitants) of psychiatric inpatient care days have declined faster than the number of patients needing psychiatric in-hospital care during the last 25 years. During the index year (1996) the number of psychiatric care days per 1000 inhabitants was 441.5 and the number of patients per 1000 inhabitants needing psychiatric in-hospital care was 6.1.

With these hospitalization patterns in mind, we calculated the yearly qualitative need for psychiatric hospital care for each individual and year with the aim to best represent an individual’s psychiatric hospital burden over time [**Fig SA3**]**.** We can show that the new metric is stable over time (**Fig SA4**), supporting that it can facilitate comparisons of psychiatric hospital need across generations.

**Fig SA3**. For each individual and each year, we asked the question: Was this person in need of hospital care primarily due to a psychiatric diagnosis (No/0 or Yes/1).


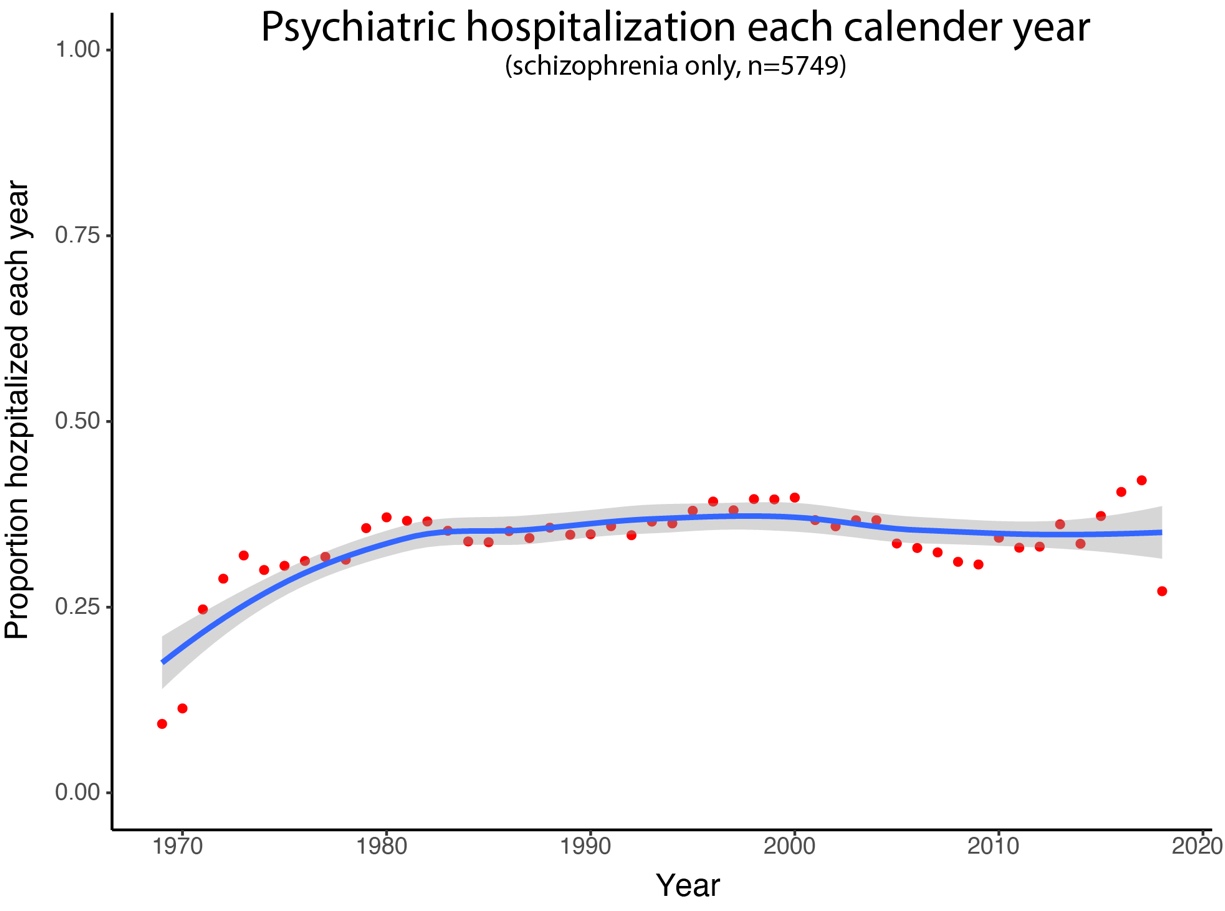


**Fig SA4.** The figure shows the proportion of individuals with schizophrenia that were hospitalized each calendar year. We see that the metric is stable over time, suggesting that our strategy is valid and that the metric facilitates comparisons between individuals, also across time. N.B. to be able to compare the hospitalization metric between time periods, we only included individuals that, at the different time points, were aged 25-35 years. [The years 1969 and 1970 are outliers, likely due to uncomplete reporting in the very beginning of the register].

**Statistical considerations**

Because of Finland’s full coverage hospital discharge registry, were >95% of true hospital visits can be identified (3), we can be confident that if an individual did not have any hospital record during a specific year, that individual was not in need of hospital care during that specific year. Thus, we did not need to treat a lack of hospital records for certain time-periods as missing data. When using the yearly hospitalization burden as the outcome in a linear regression model, we defined psychiatric hospital burden as the average yearly need for psychiatric hospital care for the first 15 years of the disease-course (counted from the age of onset of any psychotic disorder). Aligning the hospital data and setting the zero time-point to the time-point of illness onset had the advantage of normalizing for age of disease onset and allowed for inter-individual comparisons of the same disease-course stages, which we believed to be a more valid comparison. The 15-year cutoff was chosen to assess as long time-period as possible while still having complete follow-up on a majority of the study individuals (71% had at least 15 years of follow-up since illness onset). As seen in **Fig SA5**, the average yearly need for psychiatric hospital care, was adequately normally distributed for real world data.

**Fig SA5**. Histogram of average yearly hospitalization burden (0-15 years post onset of psychotic illness) within individuals with schizophrenia.

To test the robustness of the standard linear model assessing psychiatric hospitalization burden we used two alternative approaches.

**1)** A linear mixed model, where the psychiatric hospitalization status each year (the outcome) for all individuals were treated as a repeated measurement with sample-ID and time-of-measurement as random variables. [**Fig SA6**].

**2)** We also calculated the p-value from the linear regression model using permutation testing, where the dependent variable of interest (the PGS) where randomly shuffled [**Fig SA6**].

**Fig SA6.** The plot shows p-values of the association between the 7 PGSs and psychiatric hospitalization burden from 3 different statistical approaches. Test association results were similar across all statistical approaches. Due to computational reasons, 100,000 iterations were run when performing the permutation test (green), affecting the results for the EA-PGS and MDD-PGS whose p-value only could be determined to be <1-e5 (marked as *).

[LM = standard linear model, LMM = Linear mixed model, Permutation=P-value permutation test for the standard linear model]

Because the association result appeared robust across statistical approaches, we decided to keep the linear regression model for further use. We validated the use of the psychiatric hospitalization metric as a relevant disease severity measurement in **Fig 2b** (main manuscript). Also, the hospitalization burden had a better (retrospective) predictive power for identifying schizophrenia individuals within the SUPER-Finland study than the SZ-PRS had (AUC= 0.739 vs AUC = 0.671 (prediction model includes age and sex)). We therefore believe that yearly psychiatric hospitalization burden is a relevant proxy of disease severity within psychotic individuals that can be used to longitudinally assess the disease severity for different times points. This measurement also captures causes for an unsustainable living situation where the healthcare system needs to intervene, which at least from a healthcare point of view is a legitimate outcome measurement.

**Diagnostic progression towards schizophrenia**

We measured the disease progression in the psychotic spectrum as individuals who prior to their schizophrenia diagnosis, first received a diagnosis of a lower ranked psychotic disorder (SAD, BD or psychotic MDD). However, the incidence of psychotic disorders diagnoses has changed over time. This is foremost true for BD, which has increased in recent times (4), and psychotic MDD, which wasn’t even defined in ICD8 (used 1969-1986 in Finland). The changes in incidence of psychotic disorders over time likely also reflects changing diagnostic practices, which could affect the interpretation of our results. **Fig SA6** shows a comparison of the proportion of psychotic diagnosis across time between the SUPER-Finland study and Finland as a whole (FinRegisty, n = 7.2 million). FinRegisty is a nationwide registry collection that includes health registry information for all Finns alive on the 1^st^ of January 2010, as well as their parents, spouses, children, and siblings (5). The endpoint ‘psychotic MDD’ was not defined in FinRegistry and was therefore left out from the analysis. Also note that we were limited to predefined endpoints in FinRegistry and could not distinguish between BD type I and BD type II. BD type II was first introduced in DSM-IV and with the expansion of the BD spectrum, the incidence has risen markedly in recent years (4) and likely explains the large proportion of BD cases in FinRegistry after the year 2000. However, the ratio of schizophrenia vs schizoaffective disorder was similar in the two studies, as were the trend that BD has become more common also in the SUPER-Finland study.

Nevertheless, although a generational effect exists, our study results showed a strong delay (5.6 years later, p=1.02e-79) for the schizophrenia diagnosis in individuals that first received a lower ranked psychotic disorder. This observed diagnostic delay was also present across all studied decades (**Fig SA7**). Further, the study also showed that the individuals that later progressed to schizophrenia where just as sick as other schizophrenia individuals already from the start of their psychotic disease-course, prior to their schizophrenia diagnosis (i.e they needed the same amount of psychiatric hospital care). Together the data, in support of our analysis strategy, suggest that our results are not due to changing clinical practice over time, and that these lower ranked diagnoses were not set in a ‘early stage’ schizophrenia before the disorder had had time to develop further.


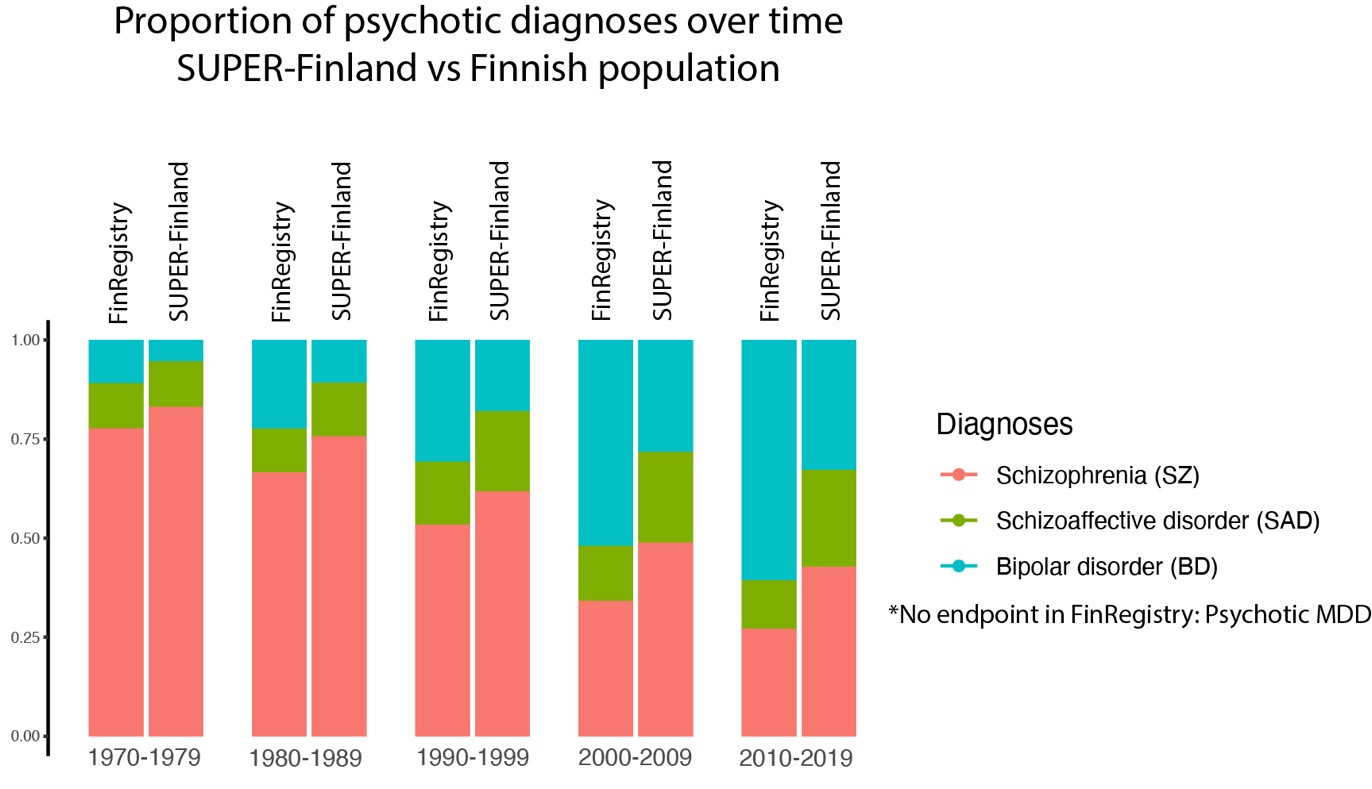


**Fig SA6.** Proportion of psychotic diagnosis set during different time-periods for the SUPER-Finland study and Finland as a whole (FinRegistry, n=7.2 million). It is known that BD spectrum diagnoses has become more common in recent years, likely explained by a larger proportion of BD type II cases. Note that the predefined endpoints in FinRegistry did not allow for trying to separate BD type I and BD type II. However, the proportion of schizophrenia cases vs schizoaffective cases between the two studies are similar. [Psychotic MDD was not defined in FinRegistry and therefore left out of the analysis.]

**Fig SA7.** The figure displays the median age of diagnosis (bars = IQR) for the four major psychotic diagnoses for each of the five decades of study follow-up. By study design, the age of diagnosis for the early time periods (note that sample size here is small) need to be low, because the individuals would have had to be young to still be alive at study inclusion (2016-2018). The group that progressed from a lower ranked psychotic disorder to schizophrenia had a later age of schizophrenia onset for all time periods, suggesting that this phenomenon is not a consequence of how clinicians choose to diagnose ‘early stage’ schizophrenia during different time periods. The number of first diagnosis for each diagnostic category and the cohorts median age (IQR) is displayed per time period (individuals not yet born during a specific time period are not counted). [Note that a single individual can have several first diagnosis (one per diagnostic group)].

**Substance use disorder (SUD) in a non-psychiatric**

**population in FinnGen.**

**The EA-PGS displays the same relationship with substance use disorder (SUD) in individuals without pronounced psychiatric illness**

Given that EA-PGS and SZ-PGS were uncorrelated (r= -0.017), it is likely that they reflect different underlying biology. For this reason, we wanted to investigate whether the EA-PGS displayed the same relationship with SUD in individuals without schizophrenia and other psychotic disorders. For this purpose, we took advantage of the FinnGen study (6), data freeze 9 (total n=377 277). The analysis revealed that the EA-PGS was strongly and consistently associated with the SUD-endpoint, regardless of cohort selection, while the results for the SZ-PGS were mainly dependent on the selected cohort’s diagnostic composition [**Fig SA8**]. Since FinnGen includes a mix of population-based cohorts and hospital-based cohorts, the biobank is enriched for disease endpoints. Performing the analysis in the whole FinnGen (n=377 277), the SZ-PGS show a strong association to the SUD-endpoint. However, when restricting the analysis to the population-based cohorts FINRISK (7) and H2000 (8) (n=34 631) the effect size of the SZ-PGS is significantly attenuated. Further, when excluding all FINRISK/H2000 individuals with a pronounced psychiatric illness, defined as having been hospitalized primarily due to a psychiatric diagnosis and/or having received a psychotic diagnosis [F20-29, F31, F32.3, F33.3], the association between the SZ-PGS and the SUD-endpoint barley reached significance (OR = 1.07 [1.00-1.15], p=0.049, n = 30 544) [**Fig SA8**].

The SZ-PGS was a good discriminator of individuals in FinnGen with a psychotic disorder (F20-29, F31, F32.3, F33.3), also when schizophrenia where excluded (OR=1.51 [1.49-1.54], p= <2.2-e308). When analyzing the full FinnGen cohort, this induced an association between the SZ-PGS and the SUD-endpoint due to a higher co-occurrence in psychiatrically ill individuals [**Table SA1**]. In contrast, the effect size for the EA-PGS remained close to unchanged when analyzed in the different sub-cohorts and its association with the SUD-endpoint was not affected by the diagnostic composition of the different cohort selections. Together the results support the interpretation that the EA-PGS have a direct effect on the risk of acquiring SUD, while the SZ-PGS does not. The results further support the hypothesis that the EA-PGS’s effect on psychiatric hospitalization burden is partly mediated via the SUD-endpoint, and independent of the SZ-PGS.

Both in FinnGen as a whole and in the two population-based cohorts (FINRISK and H2000) the SUD-endpoint showed very strong associations to the event of having needed hospital care primarily due to a psychiatric diagnosis, also after schizophrenia was excluded (OR=22.1 [21.4-22.8] and OR=22.4 [20.2-24.9], respectively). However, in individuals without a pronounced psychiatric disorder, this association could not be tested due to the cohort selection criteria.

**Associations between substance use disorder (SUD) and PGSes in FinnGen**

**Fig SA8.** Forest plot of the associations between the SUD and the EA-PGS (blue) and the SZ-

PGS (red) in three cohorts with different prevalence of psychiatric disorders. The effect size for the association between the SUD-endpoint and the EA-PGS remains close to unchanged in the 3 different cohort selections and was not dependent on the cohort’s diagnostic composition. However, for the SZ-PGS, the associations seemed to mainly be driven by the diagnostic composition of the sub-cohorts and the association was greatly attenuated when individuals with a psychiatric illness were removed.

**Table SA1. SUD prevalence in the analyzed cohorts**

| Cohort | SUD-endpoint prevalence |
| --- | --- |
| FinnGen | 6.4% |
| FINRISK and H2000 | 6.2% |
| FINRISK and H2000 (psychiatric illness excluded) | 2.7% |
|  |  |
| Schizophrenia | 32.1% |
| Psychotic disorders (excluding SZ) | 29.2% |

**References**
