# Supplementary material for: Genetic contribution to disease-course severity and progression in the SUPER-Finland study, a cohort of 10,403 individuals with psychotic disorders
Source: Mol Psychiatry. 2024 Apr 1;29(9):2733–41. doi: 10.1038/s41380-024-02516-6 (PMC11420086; doi:10.1038/s41380-024-02516-6)
Supplement: Supplementary file 3 — Supplementary Figures [file 41380_2024_2516_MOESM3_ESM.pdf]

Supplementary Figures

Fig S1

Analyses Flowchart - Overview

SUPER-Finland psychosis study
n=10 403

Genotype QC and filtering (passed: n=9 826)

Hierarchical ranking

Schizophrenia (n=5 479)

Schizoaffective disorder (n=874)

Bipolar disorder (n=1 494)

Psychotic MDD (n=507)

(Other, n= 1067 + 405)

Protocol ranked severity

For each individual, a hospitalization burden metric was calculated from the in-hospital registry (up to 50 yrs of follow-up)

Diagnosis specific psychiatric hospital burden (group level)

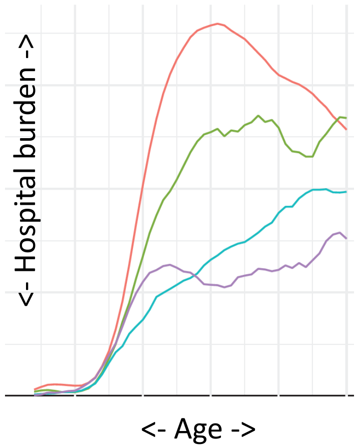

(Fig 2a in main manuscript)

Analysis 1

Focus: Progression from lower ranked psychotic disorders to schizophrenia (n=926)

Genetic contribution to psychotic diagnostic progression and hospitalization patterns towards progression.

Analysis 2

Focus: Disease-course severity in individuals with schizophrenia (n=5 479)

Genetic contribution to disease-course severity, proxied by hospital burden.
(Confided to schizophrenia to avoid diagnosis bias)

Fig S2

Progression from an initially lower ranked psychotic diagnosis to schizophrenia – Psychiatric hospitalization burden

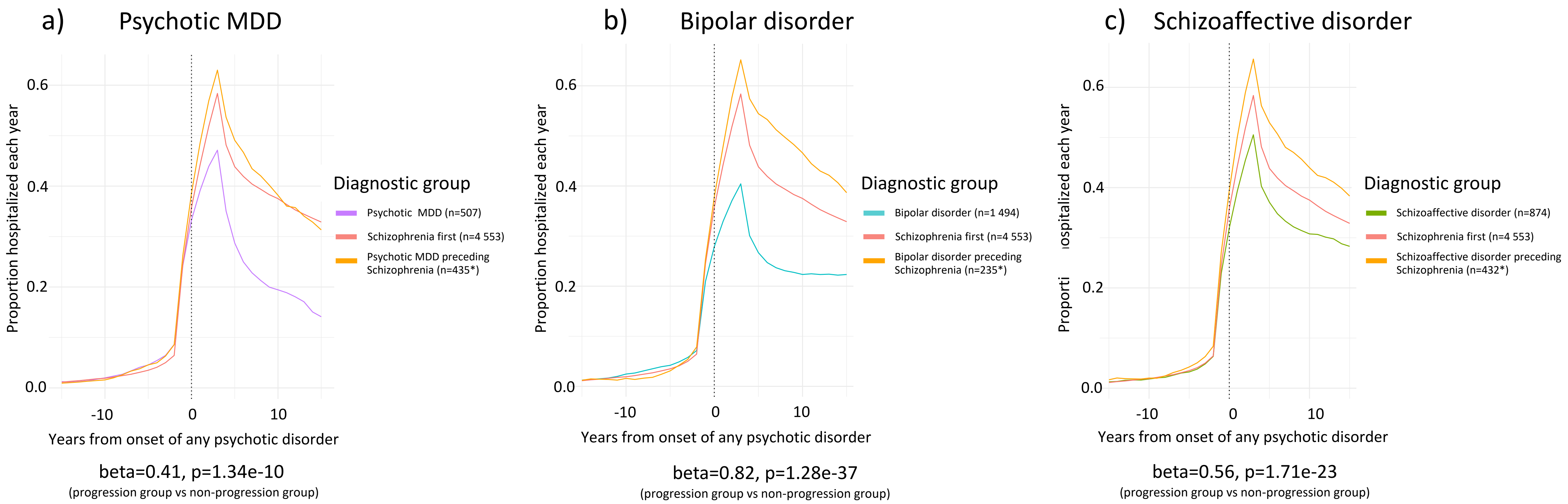

Fig S2. The psychiatric hospitalization burden for individuals that progressed to schizophrenia from an initial lower ranked psychotic diagnosis was compared to 1) The individuals that remained at the corresponding lower ranked diagnoses and 2) Individuals that had schizophrenia as their first major psychotic diagnosis (used as a reference). We found that progression groups had at least as high hospitalization burden as individuals with schizophrenia, counted from the first recorded onset of psychotic illness, and significantly higher than the corresponding lower ranked diagnoses (combined analysis: beta=0.59, p=6.09e-63). [*176 individuals overlap the progression groups, in total 926 individuals progressed to schizophrenia from an initially lower ranked psychotic diagnosis.]

Fig S3

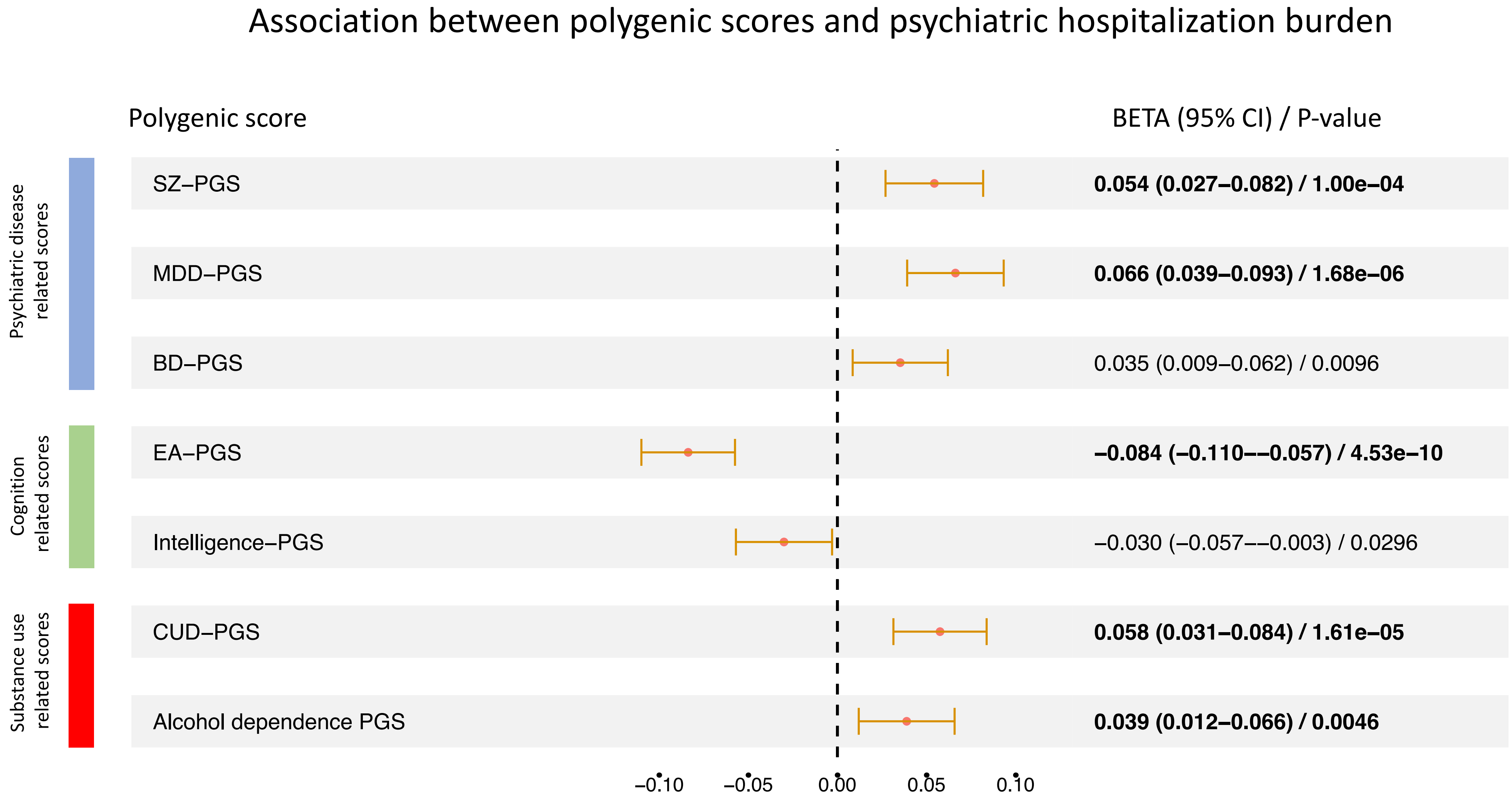

Fig S3. Polygenic scores and their association to the psychiatric hospitalization burden counted from first record of psychotic illness onset and 15 years forward. P-values that are bolded are significant after adjusting for multiple testing (p<0.05/7).

Fig S4

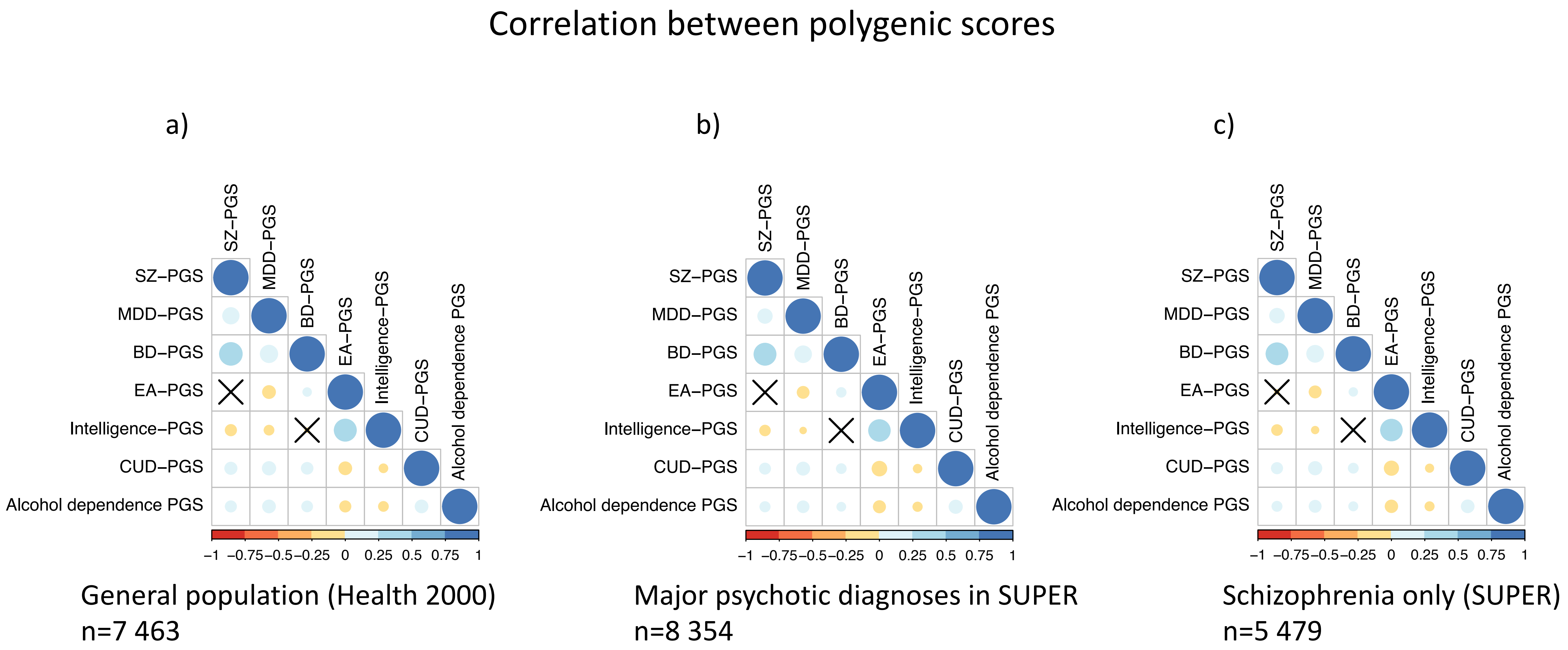

Fig S4. Panels a-b show correlation plot of all seven assessed PGSs in two different subsets of individuals with a psychotic disorder and population controls. In all three groups the SZ-PGS and the EA-PGS were independent from each other, also on a nominal significance level (i.e p>0.05). [X denotes a non-significant correlation (the significance level was adjusted for multiple testing)]

Fig S5

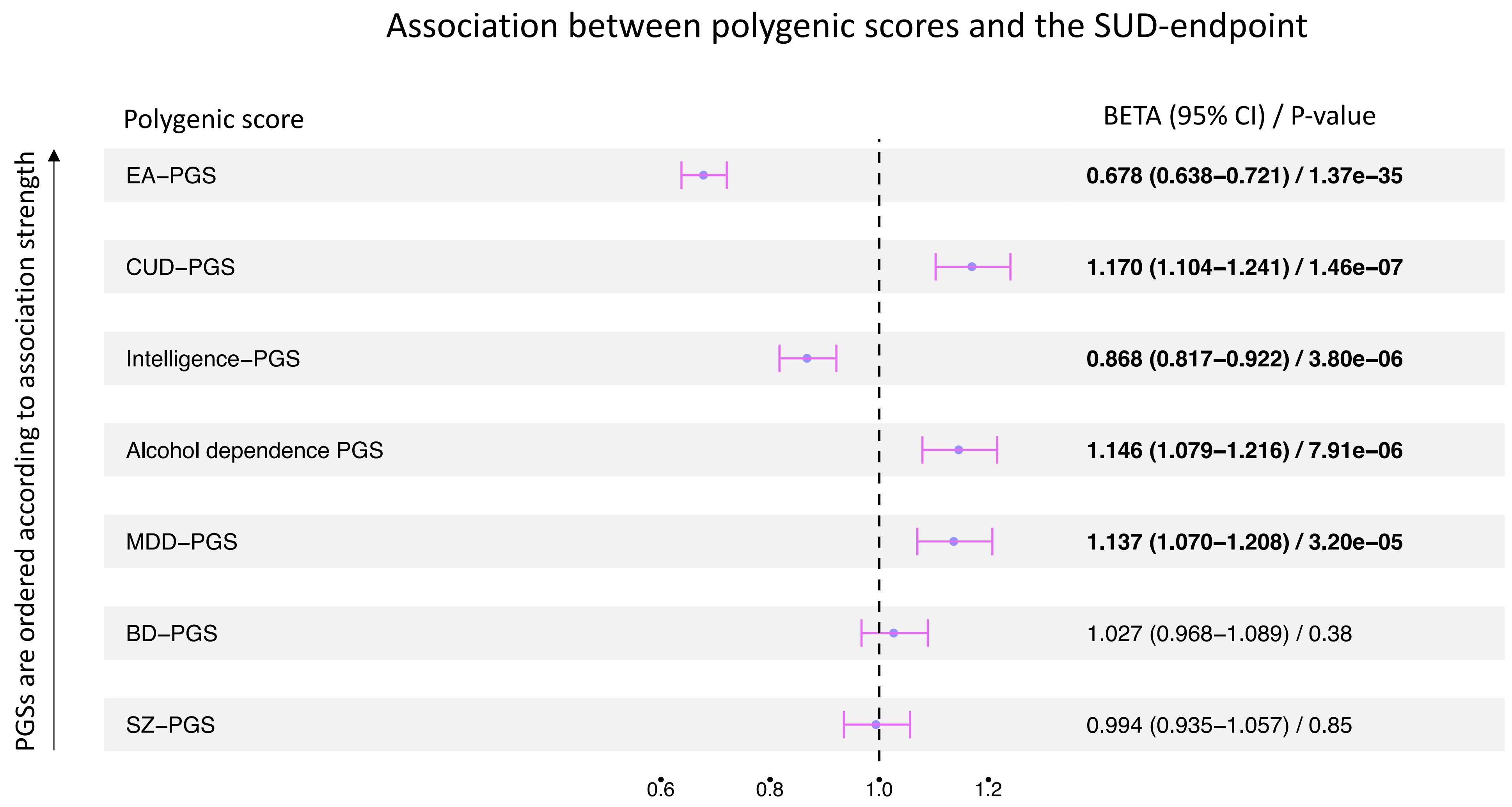

Fig S5. Polygenic scores (PGSs) and their association to the SUD-endpoint. PGSs are ordered according to association strength. P-values that are bolded are significant after adjusting for multiple testing ($p<0.05/7$).

Fig S6

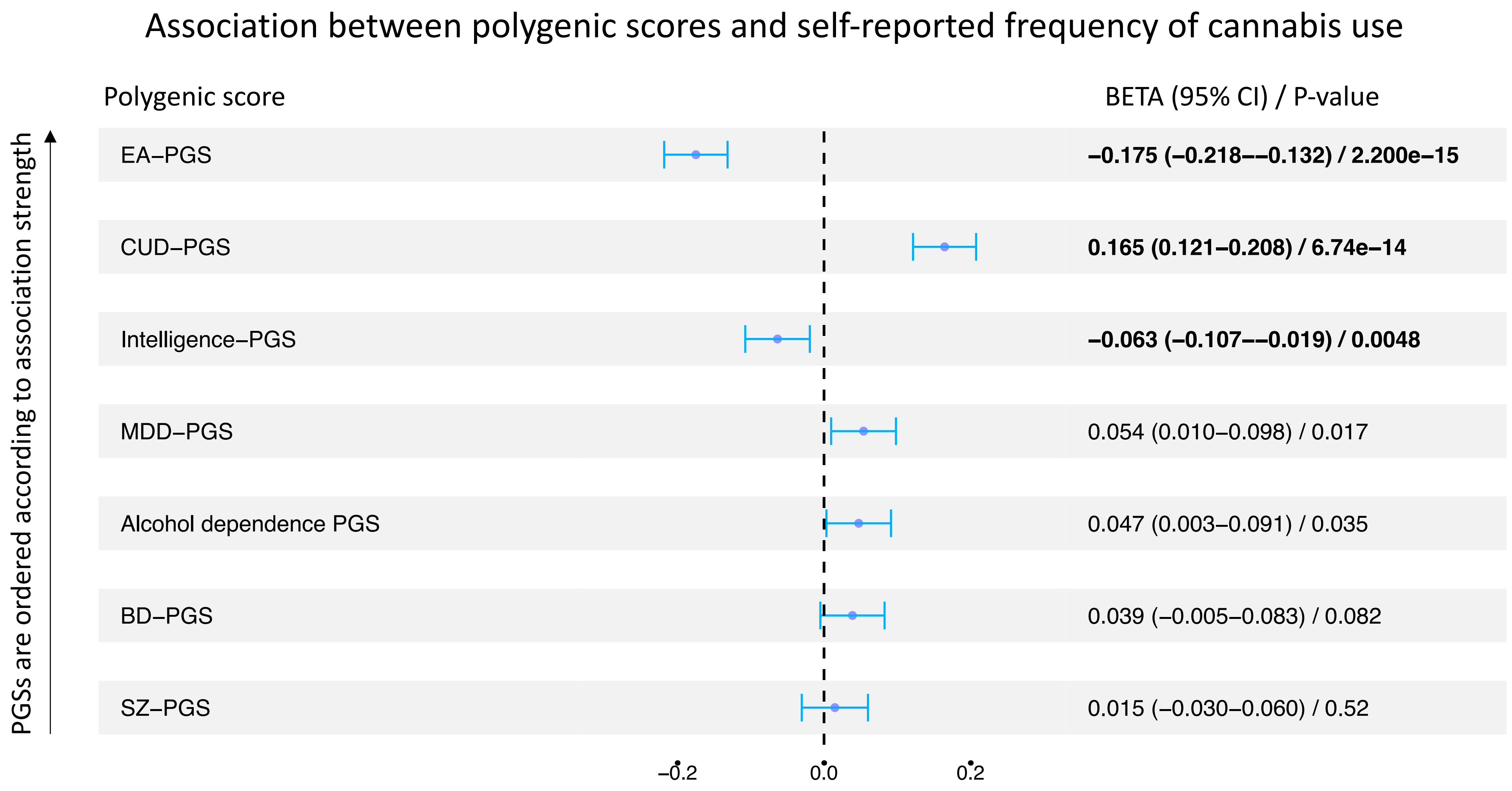

Fig S6. Polygenic scores (PGSs) and their association to self-reported frequency of cannabis use. PGSs are ordered according to association strength. P-values that are bolded are significant after adjusting for multiple testing ($p<0.05/7$).

Fig S7

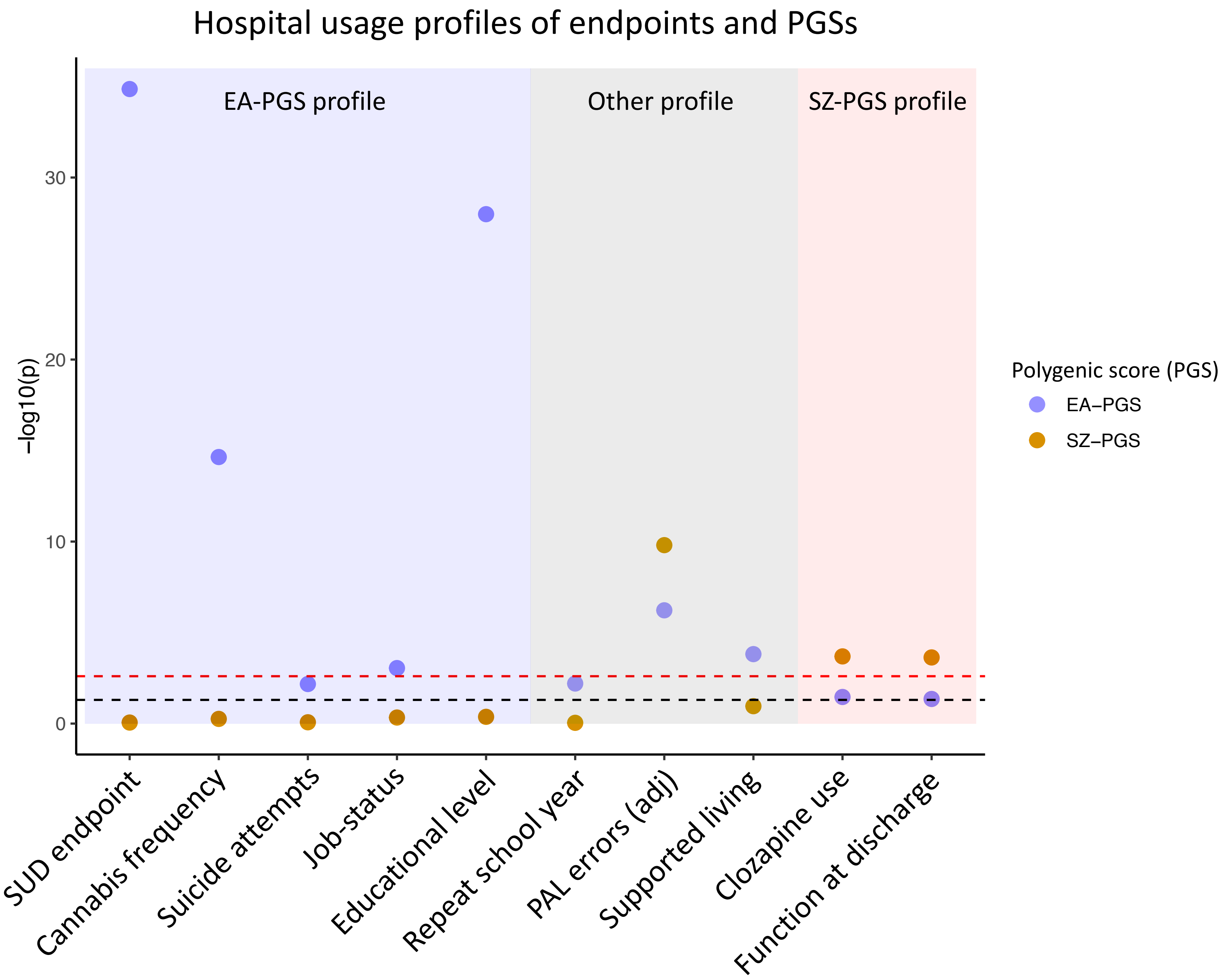

Fig S7. The graph display the association strength (p-value, y-axis) between endpoints and polygenic scores (EA-PGS and SZ-PGS [colored]). The background color show which hospital usage profile the endpoint associates with. The results show that endpoints primarily associated with the EA-PGS tend to share the same hospitalization usage profile as the EA-PGS, and vice versa for the SZ-PGS. [The red line denotes the significance level after multiple testing adjustment and the black line denotes a nominal significance level of p=0.05.]

Fig S8

Psychiatric hospitalization burden and substance use disorder (SUD)

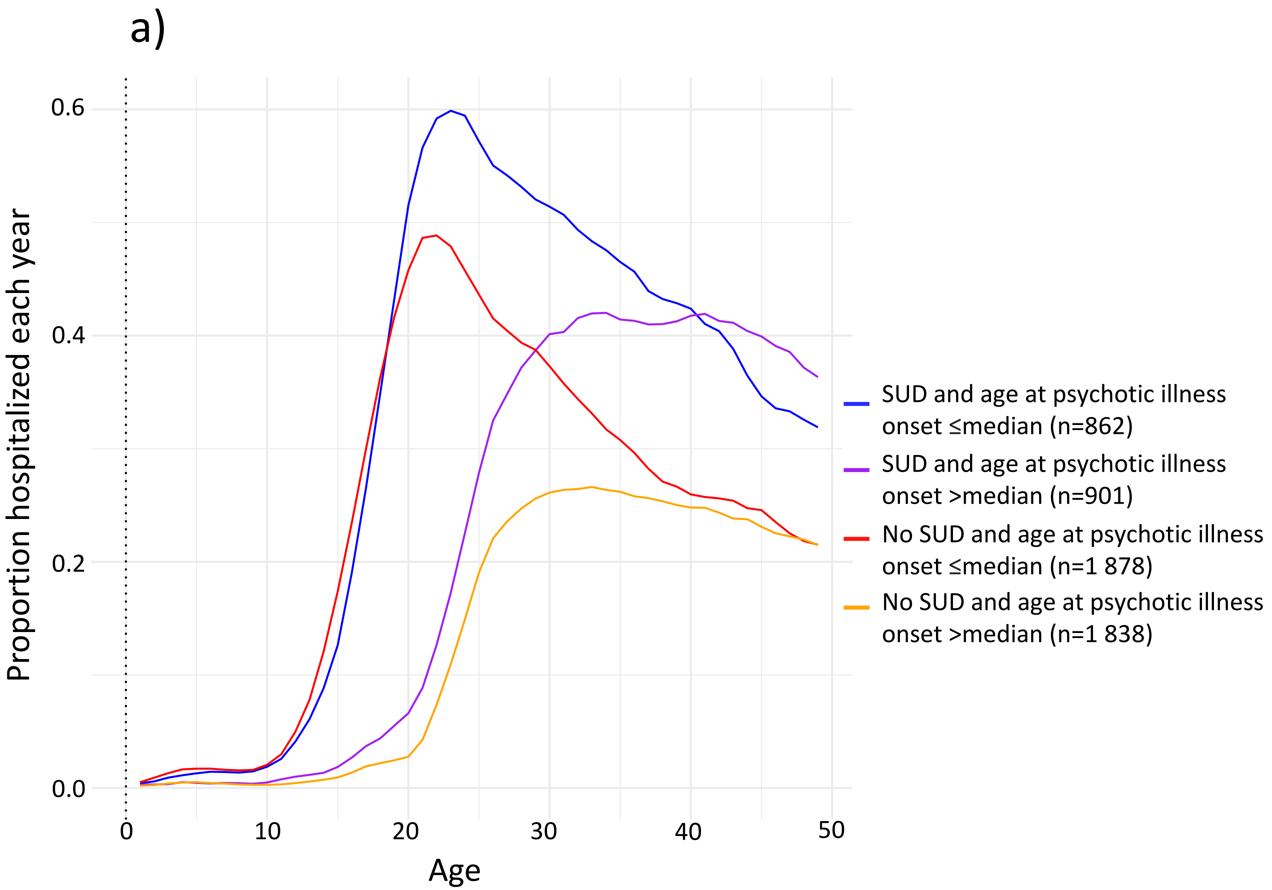

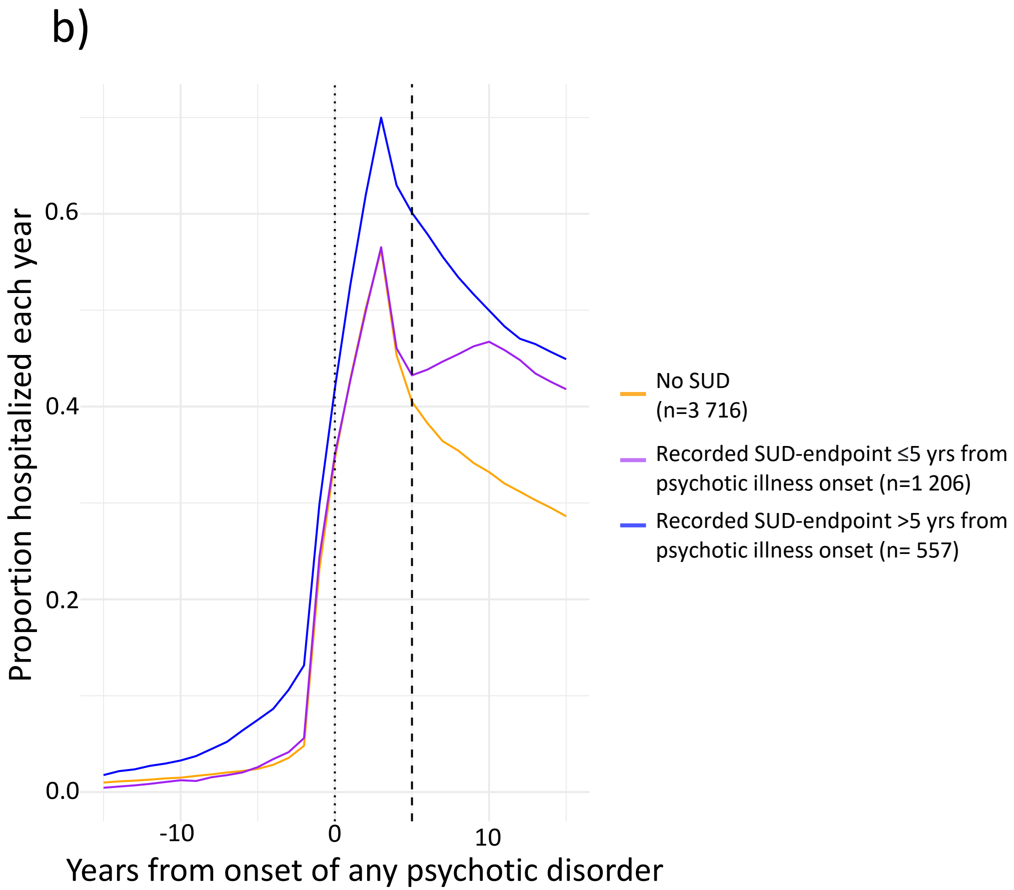

Fig S8 a). Psychiatric hospitalization burden and SUD-endpoint status in individuals with either an earlier or a later age of psychotic illness onset. Having a substance use disorder (SUD-endpoint) increased the need for psychiatric hospitalizations in both groups, but in a time-dependent manner, where age at peak hospitalization burden were dependent on age of psychotic illness onset. Thus, the endpoint did not seem to be just a marker for a poor outcome, in contrast to acquired educational level [**Fig S11**]. [Median age of psychotic illness onset was 24.4 years]

Fig S8 b). Psychiatric hospitalization burden for individuals with and without the SUD-endpoint. Individuals with the SUD-endpoint were divided into two groups based on the time-point for the first recorded SUD diagnosis. We see that the increase in hospitalization burden on average co-occur with the time-point of the first SUD diagnosis. The trend supports a direct effect of the SUD-endpoint on psychiatric hospitalization burden.

Fig S9

Association between the EA-PGS and psychiatric hospitalization burden – P-value and effect size distribution after randomly removing 1 763 individuals.
(Simulated 10 000 times)

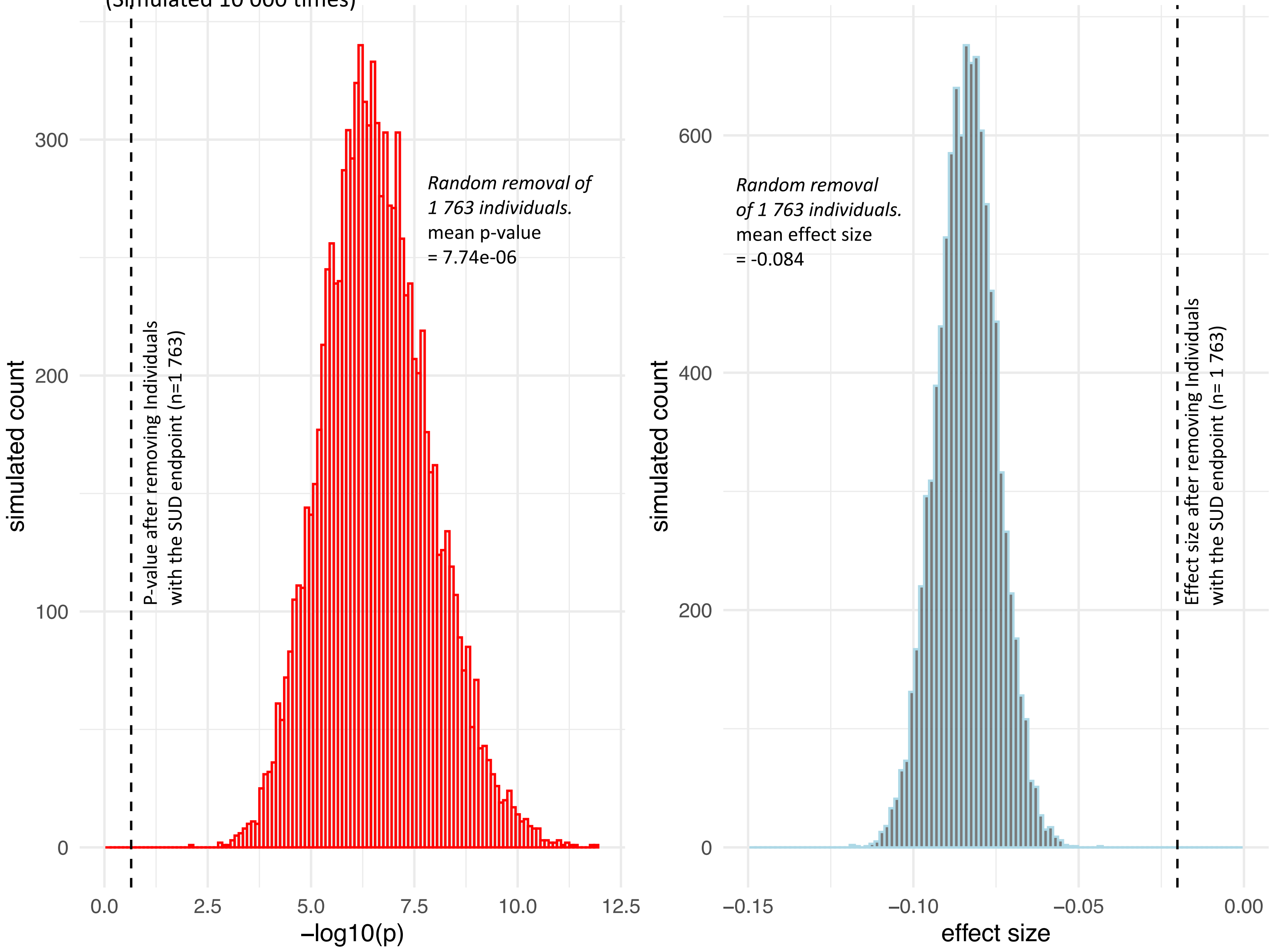

Fig S9. Distribution of p-values and effect sizes for the association between the EA-PGS and psychiatric hospitalization burden when 1 763 individuals were randomly removed (simulated 10 000 times). The dashed lines represents the actual values when the 1 763 individuals with the SUD-endpoint were removed (beta = -0.020, p=0.23). Thus, removing the individuals with the SUD-endpoint significantly attenuated the association (p<0.0001), and the EA-PGS was then no longer associated with psychiatric hospitalization burden. When randomly removing 1 763 individuals the effect size remained the same as in the original analysis (-0.083 vs -0.084).

Fig S10

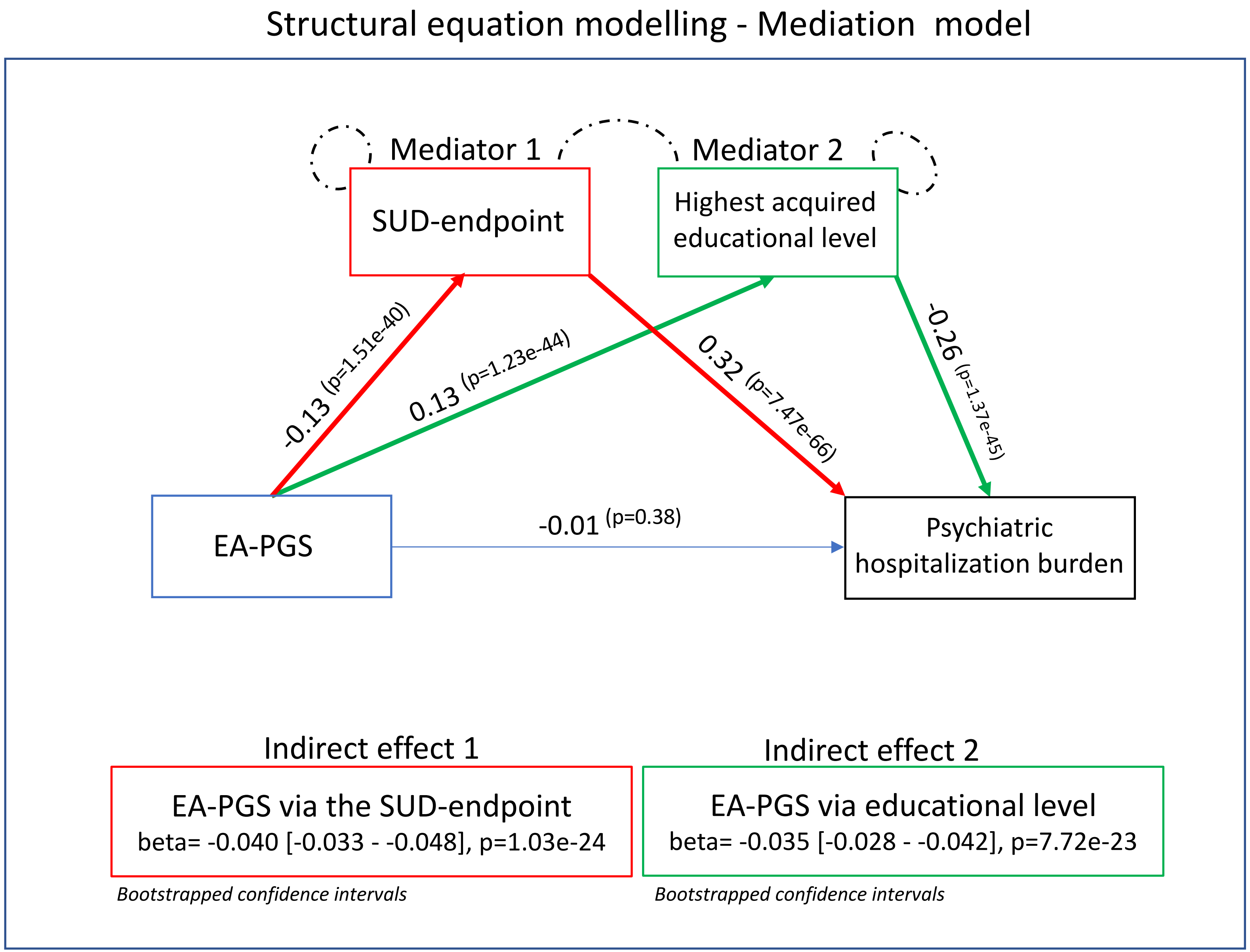

Fig S10. The mediation model includes both the SUD-endpoint and highest acquired educational level as potential mediators of the EA-PGS's effect. The results show that the EA-PGS's effect on psychiatric hospitalization burden is partly medicated via its effect on the risk of acquiring a substance use disorder (SUD). Causality is not tested, but the direction of effect is plausible. The mediated effect of the EA-PGS only slightly increased if the highest acquired educational level was excluded from the model (beta= -0.044, p=1.66e-27).

Fig S11

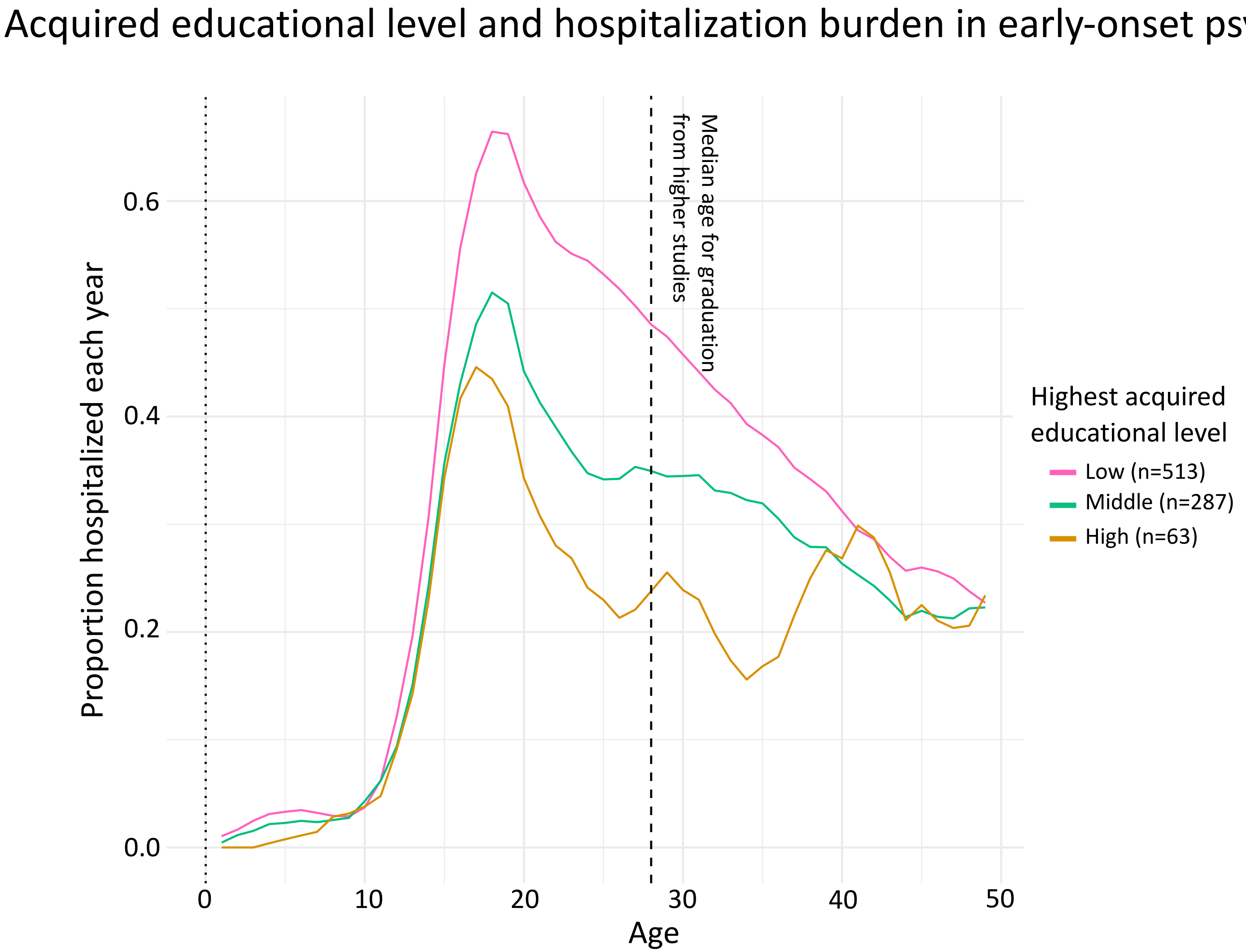

Fig S11. Psychiatric hospitalization burden in individuals with an early age of psychotic illness onset (<19 yrs) and available data on highest acquired educational level. Despite being too young at illness onset to have reached either the 'Middle' or the 'High' educational category, the future educational level still had a large impact on the hospitalization burden in young age. The observation suggests that the highest acquired educational level is foremost a marker for disease-course outcome but does not have a direct effect on hospitalization burden (compare to **Fig S8)**. [Educational levels: 'Low' = less than secondary school diploma; 'Middle' = secondary school diploma (reached at 19 yrs); 'High' = degree from university or similar (In 2010 the median age in Finland was 28 yrs)]
